# Principles and Applications of ZnO Nanomaterials in Optical Biosensors and ZnO Nanomaterial-Enhanced Biodetection

**DOI:** 10.3390/bios14100480

**Published:** 2024-10-06

**Authors:** Marion Ryan C. Sytu, Jong-In Hahm

**Affiliations:** Department of Chemistry, Georgetown University, 37th & O Sts. NW., Washington, DC 20057, USA

**Keywords:** ZnO nanomaterials, ZnO biosensors, ZnO optical sensors, optical biosensors, optical biodetection, nanobiosensors, ZnO nanoparticles, ZnO nanorods, ZnO-enhanced biodetection

## Abstract

Significant research accomplishments have been made so far for the development and application of ZnO nanomaterials in enhanced optical biodetection. The unparalleled optical properties of ZnO nanomaterials and their reduced dimensionality have been successfully exploited to push the limits of conventional optical biosensors and optical biodetection platforms for a wide range of bioanalytes. ZnO nanomaterial-enabled advancements in optical biosensors have been demonstrated to improve key sensor performance characteristics such as the limit of detection and dynamic range. In addition, all nanomaterial forms of ZnO, ranging from 0-dimensional (0D) and 1D to 2D nanostructures, have been proven to be useful, ensuring their versatile fabrication into functional biosensors. The employment of ZnO as an essential biosensing element has been assessed not only for ensembles but also for individual nanomaterials, which is advantageous for the realization of high miniaturization and minimal invasiveness in biosensors and biodevices. Moreover, the nanomaterials’ incorporations into biosensors have been shown to be useful and functional for a variety of optical detection modes, such as absorption, colorimetry, fluorescence, near-band-edge emission, deep-level emission, chemiluminescence, surface evanescent wave, whispering gallery mode, lossy-mode resonance, surface plasmon resonance, and surface-enhanced Raman scattering. The detection capabilities of these ZnO nanomaterial-based optical biosensors demonstrated so far are highly encouraging and, in some cases, permit quantitative analyses of ultra-trace level bioanalytes that cannot be measured by other means. Hence, steady research endeavors are expected in this burgeoning field, whose scientific and technological impacts will grow immensely in the future. This review provides a timely and much needed review of the research efforts made in the field of ZnO nanomaterial-based optical biosensors in a comprehensive and systematic manner. The topical discussions in this review are organized by the different modes of optical detection listed above and further grouped by the dimensionality of the ZnO nanostructures used in biosensors. Following an overview of a given optical detection mode, the unique properties of ZnO nanomaterials critical to enhanced biodetection are presented in detail. Subsequently, specific biosensing applications of ZnO nanomaterials are discussed for ~40 different bioanalytes, and the important roles that the ZnO nanomaterials play in bioanalyte detection are also identified.

## 1. Introduction

Zinc oxide (ZnO) nanobiosensors represent a cutting-edge research area which recently garnered significant technological interest due to their outstanding performance in quantitatively detecting a wide variety of bioanalytes. Extensive research efforts have proven the outstanding usefulness of ZnO nanomaterials as key signal-enhancing elements in biosensing [1,2,3,4]. Such use of ZnO nanomaterials has been demonstrated for all mechanisms of biodetection, which include optical, electrochemical, electrical, gravimetric, and magnetic signals. Among these, optical detection modes constitute one of the most widely employed methods in biology and medicine, fields in which the involvement of ZnO nanomaterials has brought significant progress. Hence, the biosensors covered in this review are focused on the intersecting research areas of ZnO nanomaterials and optical signal detection. The signal transduction modes covered are absorption, colorimetry, fluorescence, near-band-edge emission, deep-level emission, chemiluminescence, surface evanescent wave, whispering gallery mode, lossy-mode resonance, surface plasmon resonance, and surface-enhanced Raman scattering. The different optical modes of the ZnO nanomaterial-based biosensors comprehensively discussed in this review are tabulated in Table 1. ZnO nanomaterials presented in the review are categorized as 0-dimensional (0D), 1D, or 2D based on their dimensionality. ZnO nanostructures of similar shapes and sizes are often found with different names in the literature. For example, nanowires, nanobelts, nanoneedles, nanowhiskers, nanoribbons, nanorods, etc., have been used to indicate long, thin, 1D ZnO nanomaterials with typical aspect ratios of greater than 10 in length to width. For simplicity and clarity, ZnO nanostructures in this review are referred to as nanoparticles (NPs) for 0D, nanorods (NRs) for 1D, and thin films for 2D.

ZnO nanomaterial-based optical biosensors have been shown to exhibit excellent detection characteristics in terms of their sensitivity, selectivity, dynamic range, reproducibility, stability, portability, flexibility, multiplexity, and throughput. The two important considerations for sensor performance, in general, are sensitivity, i.e., the ability to show a response per unit change in analyte concentration, and selectivity, i.e., the capability of discriminating between different analytes. The sensitivity is associated with the lowest analyte concentration, called the limit of detection (LOD), that can be detected for the sensor to show a measurable response. In this review, these improvements in sensitivity and selectivity, as well as other performance characteristics mentioned earlier, will be discussed for various ZnO nanobiosensors. The enhanced performances of ZnO nanomaterial-based optical biosensors stem from the extraordinary properties of the nanomaterials that can be modified by their dimensionality, crystallinity, size, shape, aspect ratio, and morphology. The unique optical and other pertinent properties of ZnO nanomaterials exploited for various optical sensing applications are detailed under each section of the optical detection mode discussed in this Review.

## 2. ZnO Nanomaterial Biosensors Based on Different Optical Detection Modes

Table 2 outlines the different ZnO nanomaterial-based optical biosensors demonstrated for detecting various bioanalytes as listed, and it serves as an overview of this comprehensive section. For each bioanalyte discussed in this section, the table summarizes concisely the types of ZnO nanomaterials, optical detection modes employed, the roles of ZnO nanomaterials in facilitating optical biosensing, and key sensor characteristics, such as linear/dynamic detection ranges and limits of detection. Detailed discussions of the biodetection endeavors will follow the table in the order of the optical detection modes and the dimensionality of the ZnO nanomaterials, as specified in Table 1.

### 2.1. Absorption and Colorimetry

#### 2.1.1. Overview of Absorption and Colorimetry in Biodetection

Absorption and colorimetry represent the most traditionally relied-upon modes of biodetection, which typically involve a solution-phase rather than a solid-state assay. In general, the reagent volumes and analyte concentrations required in solution-phase, absorption, and colorimetry detection are considered to be higher than those needed in compact, solid-state detection platforms. However, these methods have the advantage of relatively simple instrumental needs. Bioanalyte detection can be straightforwardly carried out by using a UV–visible (UV-Vis) spectrometer for absorption or even directly by the naked eye in the case of colorimetry. This can further enable the incorporation of absorption- and colorimetry-based sensors into field-ready, point-of-care monitoring devices. In principle, the two biodetection methods can be label-free when a chromogenic bioanalyte is targeted. However, most bioanalytes are colorless, under which circumstances the measurement requires the use of external chromogenic compounds. The bioanalyte concentrations in these cases are indirectly determined by measuring those of the chromogenic compounds rather than the bioanalytes. For example, biosensors based on an enzyme-linked immunosorbent assay (ELISA) often involve the oxidation of a chromogenic substance, 3,3′,5,5′-tetramethylbenzidine (TMB), by H_2_O_2_ that is catalyzed by horseradish peroxidase (HRP) [56]. Similarly, many biosensors for enzymatic oxidation contain a bioanalyte that generates H_2_O_2_ as a by-product to trigger the oxidation reaction of an external chromogen. For bioassays involving TMB, the color change from clear to blue serves as the optical signal whose intensity is proportional to the concentration of the bioanalyte.

#### 2.1.2. Contributions of ZnO Nanomaterials in Absorption- and Colorimetry-Based Biosensors

Both 0D and 1D forms of ZnO nanomaterials have been developed to facilitate absorption- and colorimetry-based bioanalyte detection [5,7,8,10]. The use of ZnO nanomaterials aimed to promote absorption- and colorimetry-based detection by functioning as a substitute for an external enzyme needed for a chromogenic reaction. For example, ZnO nanomaterials showing an intrinsic catalytic activity similar to that of HRP for the TMB oxidation reaction can eliminate the need for an external enzyme [8,56]. In addition, the use of ZnO nanomaterials in absorption and colorimetric bioanalyte measurements aimed to promote better recruitment of biomolecules to the active sensing area of a detection platform for a faster reaction time and effective signal generation [8].

#### 2.1.3. Applications of 0D ZnO Nanomaterials in Absorption and Colorimetry Biosensors

Nanocomposites of ZnO NPs have been synthesized for absorption- and colorimetry-based detection of lactate, cholesterol, and coronavirus disease 2019 (COVID-19) virus [5,7,8]. In one study, ZnO NPs were first made into nanocomposites of Fe_2_O_3_-ZnO NPs of ~15–18 nm in size by co-precipitating the NPs from the precursor solution of Fe_2_Cl_3_·6H_2_O and Zn(O_2_CCH_3_)_2_·2H_2_O in alkaline conditions [5]. A lactate sensor of Fe_2_O_3_-ZnO NPs was constructed by adding the nanocomposites to a solution of lactate oxidase (LOx) and lactate of various concentrations. LOx oxidized lactate to pyruvate and H_2_O_2_. It turned out that, without the use of HRP, the Fe_2_O_3_-ZnO nanocomposites exhibited a peroxidase-like property and catalyzed the oxidation of TMB by the generated H_2_O_2_. The absorbance of the assay solution measured was proportional to the lactate concentration, showing a linear response in the range of 50–1000 µM with a limit of detection (LOD) of 9.4 µM. In another study, sol-gel-grown ZnO NPs of ~20 nm in size were utilized in an absorption-based COVID-19 sensor. The sensor contained a nanohybrid of chitosan film (ChF), ZnO NPs, and carbon nanotubes (CNTs) that served as a peroxidase mimic [7]. Each component of the nanohybrid was reported to possess a peroxidase-like property that could synergistically enhance the nanohybrid’s catalytic activity for the TMB oxidation reaction [56,57,58]. A COVID-19 aptamer was then immobilized onto the nanohybrid to further enhance the peroxidase activity of the nanohybrid. In the presence of a COVID-19 virus, the COVID-19 aptamer was detached from the ChF/ZnO/CNT nanohybrid due to its higher affinity towards the virus. As a result, the peroxidase activity of the ChF/ZnO/CNT nanohybrid was reduced, leading to a decrease in the amount of oxidized TMB, as well as the absorption signal. The ChF/ZnO/CNT COVID-19 sensor provided a linear range of 1–500 pg/mL and a LOD of 0.05 pg/mL.

The same nanohybrid was further employed for colorimetric COVID-19 detection to demonstrate a dual detection capability in absorption and colorimetry [7]. In this approach, the COVID-19 aptamer/ChF/ZnO/CNT nanohybrid was first deposited on a filter paper. The addition of the COVID-19 virus reduced the blue color intensity due to the reduced amount of oxidized TMB. The colorimetric detection resulted in a linear range of 50–500 pg/mL and a LOD of 8 pg/mL. In another effort, ZnO NPs were incorporated into the surface of CNTs for colorimetric detection of cholesterol [8]. The study used a chromogenic substrate of 2,2′-azino-bis(3-ethylbenzthiazoline-6-sulfonic acid) (ABTS) that forms a green-colored product upon its oxidation. Owing to their peroxidase-like activity, the ZnO NPs/CNTs were able to catalyze the oxidation of ABTS by H_2_O_2_ that was generated from the oxidation of cholesterol in the presence of cholesterol oxidase (ChOx). The absorbance, measured at 405 nm in the colorimetric method, increased linearly in the cholesterol concentration range of 0.5–500 nM with a LOD of 0.2 nM.

#### 2.1.4. Applications of 1D ZnO Nanomaterials in Absorption and Colorimetry Biosensors

**Figure 1:**
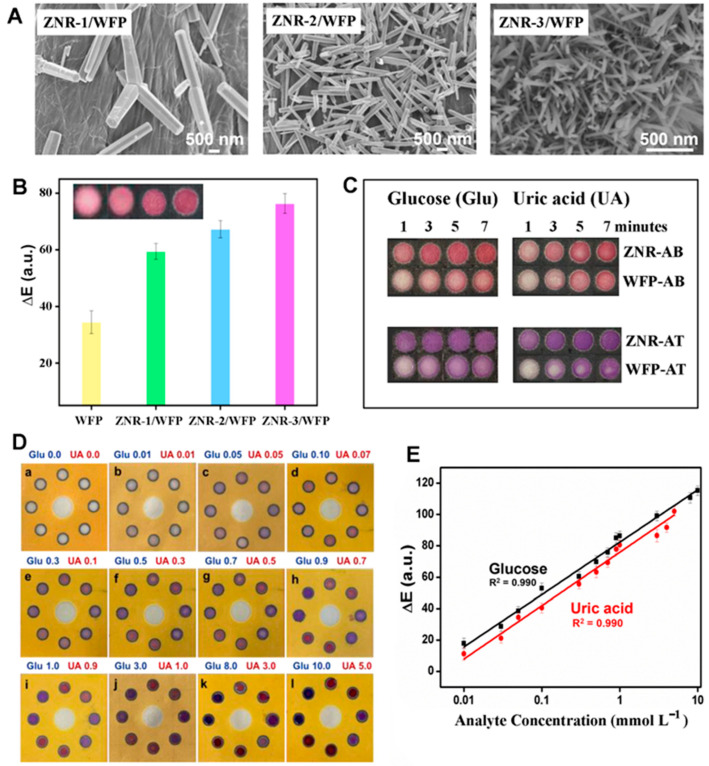
An example of a ZnO nanomaterial-based colorimetric biosensor is shown. ZnO NR-modified μPADs were developed as colorimetric biosensors for the detection of glucose and uric acid while employing the chromogenic reagents of AB and AT. (**A**) The scanning electron microscopy (SEM) panels display the ZnO NRs grown to ~5 μm in length on a Whatman filter paper (WFP). The diameters of the NRs on the μPADs are 500–700 nm for the ZNR-1/WFP, ~200 nm for ZNR-2/WFP, and ~20 nm for ZNR-3/WFP samples. (**B**) The plot displays the color differences (ΔE) observed from an unmodified WFP, as well as the three ZNR/WFP samples, when 1 mmol/L of uric acid was added to the μPAD sensor containing AB. (**C**) The colors developed on the ZnO NR-modified μPAD sensors at different incubation times are shown for the detection of both glucose and uric acid. For both analytes, the color images correspond to the case of 1 mmol/L of analyte with AB or AT reacted up to 7 min, as specified for each image. (**D**) The marked images show the detection layer of a ZNR-3/WFP device when the different concentrations of uric acid and glucose were added to the ZnO NR-modified μPAD biosensor. (**E**) The graph displays representative calibration curves obtained from the ZnO NR-modified μPAD colorimetric biosensor for the detection of uric acid and glucose. Adapted with permission from Ref. [10], copyright (2021) Elsevier B.V.

As for the use of 1D ZnO, hydrothermally grown ZnO NRs were integrated into a microfluidic, paper-based device (µPAD) and employed as a colorimetric biosensor for the simultaneous detection of glucose and uric acid [10]. Figure 1A shows the different ZnO NRs prepared on a filter paper as µPADs. The detection was based on the enzymatic oxidation of the bioanalytes, in which glucose was oxidized by glucose oxidase (GOx) to produce gluconolactone and H_2_O_2_, whereas uric acid was oxidized by urate oxidase (UOx) to form allantoin, CO_2_, and H_2_O_2_. Two chromogenic reagents of AB were selected for the glucose and uric acid assays: a mixture of 4-aminoantipyrine (4-AAP) and 3,5-dichloro-2-hydroxy-benzenesulfonic acid (DHBS); and AT, a mixture of 4-AAP and N-ethyl-N-(3-sulfopropyl)-3-methyl-aniline sodium salt (TOPS). Subsequently, GOx and UOx were deposited into different channels containing AB and AT. HRP was then added to all channels to further catalyze the oxidation of AB (red color) and AT (purple color) under the presence of H_2_O_2_ formed from the oxidation reactions of glucose and uric acid. The colors observed from the glucose and uric acid reactions on the ZnO NR-integrated µPAD are displayed in Figure 1B,C. The µPADs contained multiple reaction channels connected to a single sample reservoir to ensure the detection accuracy by the naked eye, as shown in Figure 1D. It was reported that the presence of ZnO NRs in the µPAD significantly enhanced the colorimetric signal by promoting greater enzyme immobilization and faster reaction time. Figure 1E presents representative calibration curves obtained from the ZnO NR-modified μPAD colorimetric biosensor for the detection of uric acid and glucose. The linear ranges and LODs of the ZnO NR µPAD sensor were determined to be 0.01–10 mM and 3 µM for glucose and 0.01–5 mM and 4 µM for uric acid, respectively.

### 2.2. Fluorescence

#### 2.2.1. Overview of Fluorescence in Biodetection

Fluorescence is the most widely used biodetection mechanism among the different modes of optical detection. Fluorescence represents one of the most prevalent biodetection methods, even when considering other non-optical biodetection modalities, such as electrochemical, electronic, piezoelectric, magnetic, gravimetric, and mass-sensitive methods, which are not covered in this review. Hence, fluorescence is a very important technique in the fields of biology, biophysics, biochemistry, gene profiling, proteomics, drug discovery, disease diagnostics, and clinical testing. Fluorescence is considered the technique of choice for these applications since it can provide good sensitivity to analyte components in complex biomolecular assemblies and is highly versatile in accommodating a range of sample types for investigation. Fluorescence detection is a more direct assessment and, therefore, offers higher quantitation accuracy and a broader dynamic linear range than schemes with linked enzymes. In addition, fluorescence-based methods facilitate multiplexing for the simultaneous detection of many bioanalytes. Fluorescence detection has greater infrastructural requirements than those involving film or naked eye-based light capture but, overall, at a modest level to include a specialized digital imager and different filters for multiplexing. All these factors combined contributes to the popularity of the modality in optical detection. Despite this, fluorescence-based techniques still face challenges related to the detection of low-abundance bioanalytes and the use of fluorophores. To overcome these limitations, extensive research efforts have been made to formulate better fluorophores for increased signal intensity and stability, to engineer advanced substrates for solid-state detection, to improve detection instruments using confocal and multiphoton sources, and even to eliminate the use of fluorescent labels entirely [11,59,60].

#### 2.2.2. Contributions of ZnO Nanomaterials in Fluorescence-Based Biodetection

##### 2.2.2.1. Quantum Dot Fluorescence Probes

One of the most recognizable contributions of nanoscience to fluorescence detection lies in the area of developing new inorganic fluorophores. A well-known example is semiconductor NPs, known as quantum dots (QDs), whose emission spectrum at a specific wavelength can be tuned by simply changing the size of the nanomaterials [61,62,63,64]. Numerous research efforts have been made to create QDs with high quantum yield, minimal photobleaching, prolonged biostability, and negligible biotoxicity [61,65,66,67]. As a part of this endeavor, 0D constructs of ZnO, as well as related nanomaterials such as ZnS, ZnTe, and ZnSe QDs, have been developed to circumvent the drawbacks of conventional, organic fluorophores and to yield composition- and size-dependent optical properties [68,69,70,71,72]. In addition to single-composition QDs, multicomponent QDs of a core/shell configuration have also been formulated in order to circumvent the unwanted effects of surface defects and further increase QD efficiency. The presence of a shell layer in a core/shell QD facilitates passivation of the defect-associated trap states that can hamper the radiative recombination and the QD efficiency [61,63,65]. Regardless of the different QD types, solution-based synthesis methods, such as hydrothermal, sol-gel, and co-precipitation, were typically employed to produce ZnO and related QDs for QD fluorophore applications [66,73].

Fluorophores of ZnO QDs and ZnO-related nanomaterials present distinctive advantages relative to conventional counterparts used in biosensing and bioimaging. In particular, ZnO QDs are considered to be low-cost, biocompatible alternatives to current fluorophores, as well as toxic, Cd-containing, and/or water-insoluble QDs [66,67]. The emission profiles of ZnO QDs depend on the particle size and the presence of surface defects that are highly tunable and controllable during their well-established synthesis methods [73,74]. It is well known that QDs exhibit stable, bright fluorescence with a broad excitation spectrum and a very large absorption cross-section [61,62,63,64,65]. At the same time, QDs display a tight Gaussian emission at wavelengths that can be tuned by the size of the nanomaterials without having to alter their chemical compositions. In some cases, QDs offer a large Stokes shift of more than 100 nm [61]. The broadband absorption and narrow symmetric emission of QDs mark a striking difference from the behaviors of conventional fluorophores. The QD emissions are also reported to be more photostable than those of organic fluorophores by many orders of magnitude. In addition, QDs exhibit long fluorescence lifetimes, making it convenient to separate the signals from the background autofluorescence. These properties enable the employment of multiple QDs with varying sizes in multiplexed biodetection, under which the QDs can be simultaneously excited by a single excitation source. The different emission contributions from various QDs used for multiplexing can be easily discerned from their sharp emission peaks with minimal spectral overlap.

##### 2.2.2.2. Fluorescence Signal-Enhancing Platforms

Unlike the predominant use of 0D ZnO as novel fluorophores, the applications of 1D and 2D ZnO nanomaterials have mainly been geared towards the development of fluorescence signal-enhancing and signal-directing platforms. In this regard, 1D ZnO nanomaterials, such as individual ZnO NR optical guides, have been fabricated for the highly intensified and spatially localized delivery and collection of fluorescence signals from bioanalytes. Two-dimensional ZnO thin-film structures have also been constructed into a solid-state biosensor substrate that can globally enhance the fluorescence signals in the sensor. One-dimensional and 2D ZnO nanomaterials can satisfy multiple important criteria in fluorescence-based biodetection. They include the nanomaterial’s ability for fluorescence signal enhancement, easy synthesis and simple fabrication steps for the nanomaterials, good chemical stability under common bioreaction environments, and available surface chemistry to tether bioanalytes to the nanomaterial surface. In the ZnO nanomaterials’ role as an optical waveguide or optical signal-enhancing support, the presence of ZnO should not hinder or alter the absorption and emission processes of the biomolecules under examination. The nanomaterials should not exhibit absorption or display autofluorescence in the spectral range of the fluorophores commonly used in biodetection. Hence, ZnO nanomaterials used in biodetection as fluorescence signal-guiding and -enhancing platforms should be prepared into highly crystalline, optical quality materials whose structures are well defined by their mirror-like, hexagonal facets of a wurtzite crystal without the presence of physical defects and chemical impurities.

#### 2.2.3. Applications of 0D ZnO Nanomaterials in Fluorescence-Based Biodetection

**Figure 2:**
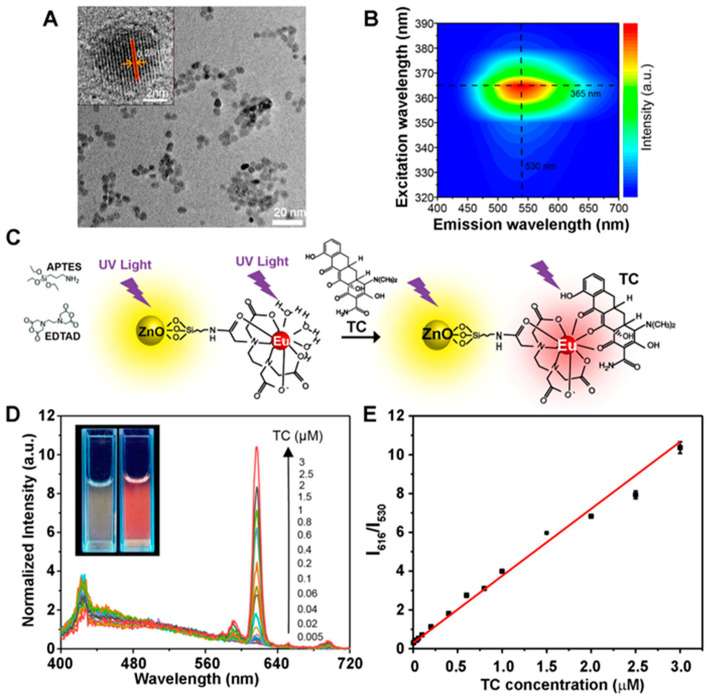
An example of ZnO nanomaterials developed as QD fluorophores is displayed. (**A**) The panel corresponds to a transmission electron microscopy (TEM) image of ~5 nm ZnO QDs used in the detection of TC. The high-resolution image in the inset shows lattice fringes at a distance of 0.28 nm due to the inter-plane distance of the ZnO (100) plane. (**B**) The excitation and emission spectra of the ZnO QDs are shown in the contour plot. (**C**) The schematic illustrates how Eu/ZnO nanostructures were constructed for TC detection. Using the chemical reaction schemes, Eu^3+^ was anchored on the surface of the Eu/ZnO QD fluorescent probe for the rare earth ion’s sensitization by TC. (**D**) The plot displays the emission spectra of the Eu/ZnO QDs under varying concentrations of TC up to 3 μM. The photographs in the inset were taken from the solution of the Eu/ZnO QDs with no TC (left) and 3 μM TC (right) under a 365 nm UV lamp. (**E**) The ratiometric calibration curve of the biosensor employing the fluorophores of Eu/ZnO QDs is shown as a function of TC concentration. The basis of the ratiometric analysis was I_616_/I_530_, which is the measured fluorescence intensity of Eu^3+^ at 616 nm divided by that of ZnO QDs at 530 nm. Adapted with permission from Ref. [18], copyright (2021) Elsevier B.V.

As discussed above, all ZnO nanomaterial forms ranging from 0D to 2D have been explored in fluorescence-based bioanalyte detection. As for 0D, various QDs containing ZnO have been explored as new inorganic fluorophores for the detection of bioanalytes such as dopamine, cysteine, DNA, avidin, calcium dipicolinate (CaDPA), and tetracycline (TC) [16,17,18,19,75]. For example, a ZnO QD-based fluorescent probe was developed for the detection of cysteine [16]. A melamine pre-probe (AMP) was synthesized by a condensation reaction between melamine and syringaldehyde, after which the AMP was mixed with sol-gel-grown ZnO QDs of ~30 nm in size. The inclusion of ZnO in the fluorescent probe of AMP/ZnO QDs aimed to generate a strong emission intensity at 482 nm, high photostability, and a large Stokes shift. The QD emission red-shifted to 516 nm in the presence of cysteine. The bathochromic shift was explained by hydrogen bonding formed between AMP/ZnO QDs and cysteine. Using the AMP/ZnO QDs, a linear range of 0.1–600 µM and a LOD of 0.642 µM were obtained from the cysteine detection. In another study, sol-gel-grown ZnO QDs coupled with a rare earth element of Eu were used as a fluorescent probe to detect CaDPA, a biomarker for anthrax [17]. The ZnO QDs were further capped with (3-aminopropyl) triethoxysilane (APTES), after which Eu^3+^ ions were chelated to the APTES-modified surface of the ZnO QDs. The Eu/ZnO QDs showed only the emission from ZnO QDs at 530 nm since rare earth ions are known to have low extinction coefficients due to forbidden 4f-4f transitions [18]. The Eu^3+^ ions in the Eu/ZnO fluorescent probe were to coordinate with DPA in CaDPA. When a Eu/ZnO-DPA complex was formed in the presence of CaDPA, DPA sensitized the emission of Eu^3+^ ions at 616 nm. The emission intensity of ZnO QDs in the complex, however, was not affected by CaDPA. Hence, the measurements were carried out by measuring the Eu^3+^ emission as the analyte signal with respect to the reference signal of ZnO QDs. The ratio of the Eu^3+^ and ZnO QD intensities (I_616_/I_530_) was found to increase linearly in the CaDPA concentration range of 0–4 µM with a LOD of 3 nM. In a different work, similarly prepared Eu/ZnO QDs were used to detect TC, yielding a linear range of 5 nM–3 µM and a LOD of 4 nM [18]. Figure 2A,B display the size and morphology of the as-grown ZnO QDs and their PL behaviors in the absorption and emission contour plot. The chemical reactions for coupling Eu^3+^ to the ZnO QDs and the coordination of TC to Eu^3+^ are illustrated in Figure 2C. Figure 2D,E show the fluorescence emission spectra, as well as the ratiometric calibration curve obtained from the Eu/ZnO QDs under varying concentrations of TC. For a portable and rapid naked-eye detection of TC, the Eu/ZnO QDs were also made into a nanocomposite, which was then deposited onto a filter paper. Under UV illumination, the color of the Eu/ZnO QD-treated paper changed from yellow to red with increasing TC concentrations. The semiquantitative determination of TC was monitored by taking the ratio of the red and green channels (R/G) of an RGB color image obtained from a smartphone. The R/G ratio showed a linear relationship in the TC concentration range of 0–30 µM with a LOD of 0.5 µM.

QDs have also been used widely in Forster or fluorescence resonance energy transfer (FRET)-based strategies [76,77,78,79]. FRET involves a non-radiative transfer of energy from an excited donor molecule to a nearby acceptor molecule in its ground state through long-range, dipole–dipole interactions [77,78,79,80,81]. The efficiency of FRET depends on multiple factors, such as the spectral overlap degree, relative dipole orientation, quantum yield, and the distance between the donor and acceptor molecules. FRET is particularly sensitive to the change in distance between the donor and acceptor molecules, with its inverse sixth power dependence on the intermolecular separation [82,83]. This strategy is exploited to detect bioanalyte binding events. FRET signals can be an accurate measure of the intermolecular separation at angstrom distances so long as the donor and acceptor molecules are positioned at a distance range of ~1–10 nm [84]. If the two molecules are separated much closer or farther than the Forster distance, the FRET signals become negligible due to quenched fluorescence and poor energy transfer, respectively. The optimal distance for highly efficient FRET measurements is ~3–6 nm of intermolecular separation, and this distance at which half the excitation energy of the donor is transferred to the acceptor is called the Forster radius [84].

A donor–acceptor FRET pair of ZnO/CdS QDs-graphene oxide (GO) was used to detect a 40-base-pair-long DNA called P_4_-DNA [19]. Layered materials were prepared on a quartz substrate from GO, T_4_-DNA (a single-stranded DNA (ssDNA) probe complementary to P_4_-DNA), and ZnO/CdS QDs. When P_4_-DNA was hybridized with the complementary T_4_-DNA, the distance between the ZnO/CdS QDs and GO was reduced due to the formation of double-stranded DNA (ds DNA). The shorter distance between the donor and acceptor promoted FRET and, hence, the binding of P_4_-DNA caused quenching of the fluorescence signal. The relative fluorescence intensity ratio with and without P_4_-DNA was found to be linearly dependent on the negative logarithm of the P_4_-DNA concentration in the range of 75.82 pM–15.28 nM, with a LOD of 8.289 pM. In another work, ZnS/ZnO QDs were employed in a time-resolved FRET (TR-FRET) detection of avidin [20]. The QD of ~4.8 nm in diameter contained a Mn-doped ZnS core capped with glutathione (GSH), after which a ZnO shell was grown around the core to reduce the surface defects and enhance the QD emission. The QD was then conjugated with biotin via the 1-ethyl-3-(3-dimethylaminopropyl) carbodiimide/N-hydroxysuccinimide (EDC/NHS) chemistry. The QD exhibited strong fluorescence at 585 nm due to the internal electronic transition ^4^T_1_ → ^6^A_1_ of Mn. In the subsequent detection of avidin, the Mn-doped ZnS/ZnO QDs and sulforhodamine 101 (SUF101)-labeled avidin served as the FRET donor–acceptor pair. The binding of SUF101-avidin to the biotinylated QDs led to the emission from SUF101 at 610 nm while quenching the QD fluorescence at 585 nm. The prolonged emission of SUF101 due to FRET with the QD donor was distinguished from the short-lived, direct excitation-induced emission of SUF101. This time-resolved detection strategy contributed to reducing the background signal. The concentration of avidin was quantified by using the intensity ratio of SUF101_610_/Mn_585_, whose values increased linearly for the avidin concentration range of 10–100 nM with a LOD of 3 nM.

#### 2.2.4. Applications of 1D ZnO Nanomaterials in Fluorescence Biosensors

##### 2.2.4.1. ZnO Nanorods in Fluorescence-Based Biodetection

ZnO NRs, owing to the reduced dimensionality and high shape anisotropy of the 1D materials, are known to exhibit subwavelength waveguiding phenomena, which can make them capable of enhancing fluorescence signals from nearby bioanalytes. This property of ZnO NRs is advantageous both for the excitation and emission processes involved in fluorescence-based biodetection [1,29,32,33,35,85,86,87,88]. The shape of ZnO NRs permits a high concentration of an electromagnetic field and, thus, an efficient excitation of bioanalytes on the NR surface. In addition, much of the excitation light exists in the form of surface evanescent waves since the widths of the ZnO NRs are smaller than those of the excitation light typically used in biodetection. Hence, undesirable background signals often associated with fluorescence detection can be minimized by exciting only those analytes linked to the surface of ZnO NRs. Moreover, ZnO NRs are well suited for spatially concentrating and waveguiding electromagnetic fields due to their high refractive index, with a value greater than 2, across the visible spectral region [89,90,91]. As for the bioanalyte signals emitted, the subwavelength waveguiding property of ZnO NRs can permit effective channeling and propagation of even a very weak signal from low-level bioanalytes.

In addition to ZnO NRs, other NR materials, such as SnO_2_, can be used in biodetection in a similar way. The nanosized diameter and the subwavelength waveguiding property of these ZnO and related materials make them suitable as minimally invasive probes to deliver light to or collect optical signals from bioanalytes in single-cell studies and in vivo imaging. The NR’s exquisite ability to illuminate, collect, and deliver light signals has already been demonstrated in subwavelength photonics [92]. For an example of a GaN NR connected to a SnO_2_ NR at one end, it was shown that the band-edge emission from the GaN NR was effectively channeled through the SnO_2_ NR to eventually emerge at the unconnected end of the SnO_2_ NR. The work additionally employed a ZnO NR, incorporating both a ZnO NR and a GaN NR connected to a SnO_2_ NR in a waveguide construct. It was revealed that optical signals can be transferred between the different NRs and be guided hundreds of µm away from their source. The cut-off wavelength of NR waveguides was dependent on both their cross-sectional dimensions and overall length. This will be useful in detecting fluorescence signals from analytes that are buried deep in a specimen matrix and hard to access for the efficient excitation and collection of their optical signals. In a proof-of-concept study, one end of a SnO_2_ NR was embedded with a droplet of a rhodamine 6G (R6G) solution, after which blue light was launched from the other end of the NR for excitation [92]. This resulted in strong fluorescence from the droplet. At the same time, a fraction of the emission was coupled to the NR and subsequently guided back to the other end of the SnO_2_ NR. When an R6G droplet was deposited at the midpoint along the NR instead, it was determined that R6G was excited by the evanescent field generated along the NR surface. Such utility of NR in fluorescence detection was later confirmed in many works directly related to biodetection, which will be discussed next.

**Figure 3:**
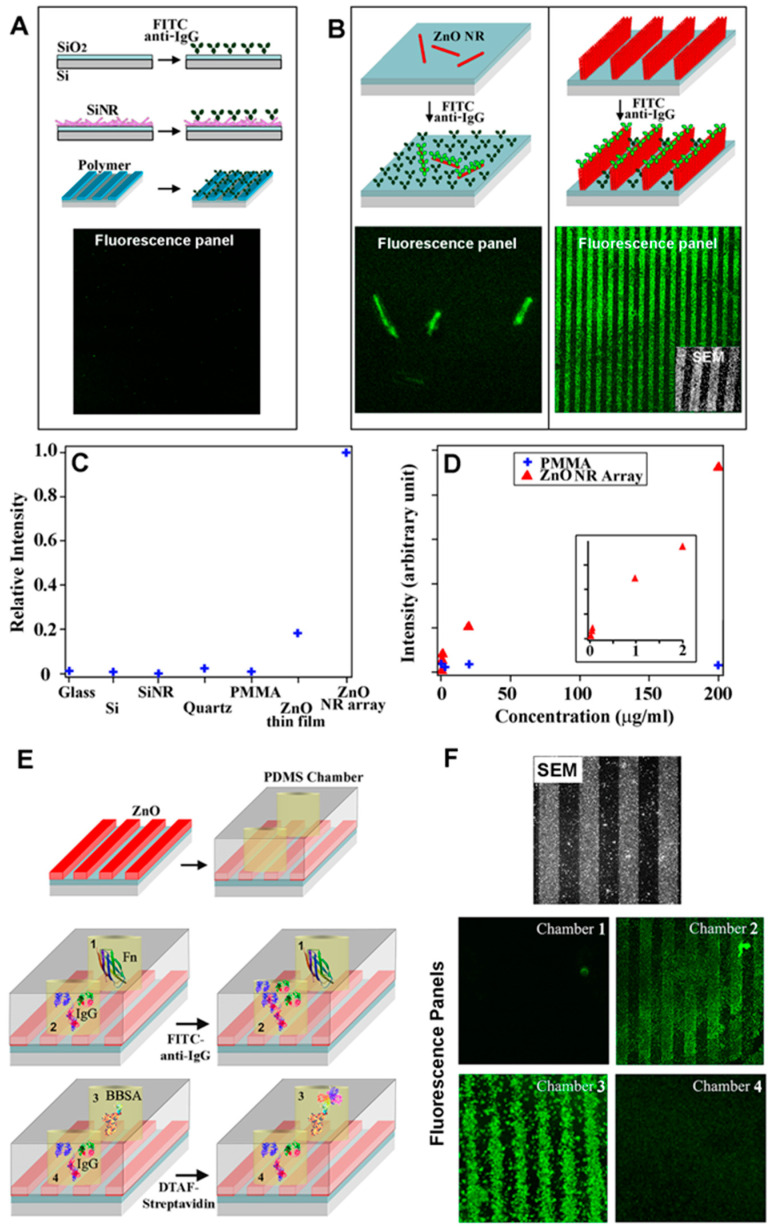
Enhanced fluorescence detection of model bioanalytes facilitated by the use of ZnO NRs is exemplified. (**A**) No significant fluorescence emission was observed from fluorescein isothiocyanate-conjugated anti-immunoglobulin G antibodies (FITC–anti-IgG) when it was adsorbed on the control substrates of Si wafers, Si NRs, and stripe-patterned PMMA substrates, even with protein concentration as high as 2 mg/mL. (**B**) Markedly strong fluorescence emission was detected from 200 μg/mL FITC–anti-IgG that were deposited on ZnO NRs grown on a Si substrate either to lay flat (left panel) or to form a striped array of vertically aligned NRs (right panel). The fluorescence images in (**A**,**B**) are 25 × 25 μm^2^ in size. The inset of the fluorescence panel in (**B**) corresponds to the top-down SEM view of the vertically grown ZnO NRs assembled into a striped array. (**C**) The plot compares the fluorescence intensity from 200 μg/mL FITC–anti-IgG molecules prepared on the different platforms of glass, Si, Si NRs, PMMA, ZnO thin film, and a striped array of ZnO NRs. (**D**) The plot of fluorescence intensity versus FITC–anti-IgG concentration compares the detection capability of ZnO NRs relative to a conventional platform of PMMA. (**A**–**D**) Adapted with permission from Ref. [21], copyright (2006) American Chemical Society. (**E**) Protein–protein interactions were performed on a striped array of ZnO NRs. The NR platform was prepared by placing an elastomeric polymer piece of PDMS that contained two hollow chambers to simultaneously carry out protein reactions on the same platform. Protein pairs examined are fibronectin (Fn) and FITC–anti-IgG, IgG and FITC–anti-IgG, biotinylated bovine serum albumin (BBSA) and dichlorotriazinylamino fluorescein-conjugated streptavidin (DTAF–streptavidin), and IgG and DTAF–streptavidin in the reaction chambers 1 through 4. (**F**) The SEM image displays the top-down view of a striped array of ZnO NRs equally present inside all reaction chambers. As seen in the fluorescence panels, strong emissions were observable due to the reactions between interacting protein pairs in chambers 2 and 3. No detectable fluorescence signals were seen from chambers 1 and 4 due to the lack of specific protein–protein interactions. (**E**,**F**) Adapted with permission from Ref. [26], copyright (2006) WILEY-VCH Verlag GmbH & Co. KGaA, Weinheim.

The fluorescence signal-enhancing phenomenon of ZnO NRs was first realized in a study comparing the fluorescence intensities of model bioanalytes assayed on different platforms, although the exact mechanism of signal enhancement was unclear at that time [21]. An array of ZnO NRs was fabricated directly upon the NR synthesis via chemical vapor deposition (CVD). A model bioanalyte of fluorescein isothiocyanate-conjugated anti-immunoglobulin G antibodies (FITC–anti-IgG) was deposited on the ZnO NR array, as well as on other control platforms of glass, quartz, Si, polymethylmethacrylate (PMMA), Si NRs, and ZnO thin film. Significantly enhanced fluorescence was monitored from FITC–anti-IgG on the ZnO NR array relative to the control platforms. The enhancement observed on the 1D ZnO NRs was also found to be greater than that on silicon nanorods (Si NRs) of similar dimensions, as well as that on a 2D ZnO thin film. The enhanced detection on ZnO NRs relative to Si NRs indicated that the enhancement was due to the inherent optical properties of ZnO rather than the inherently larger surface area of nanomaterials relative to the conventional substrates. ZnO NRs exhibited ~4-fold higher fluorescence signals than the ZnO thin film, which suggested that the reduced dimensionality of ZnO should be further considered for signal enhancement. These results are evidenced by the fluorescence data shown in Figure 3A–D. Overall, the study demonstrated for the first time that ZnO nanomaterials, particularly 1D ZnO NRs, are ideal platforms for enhancing the fluorescence signals of the bioanalytes placed in the vicinity of the ZnO NRs.

Ensuing research investigations in the next decade revealed the origin of the enhancement and its connection to the subwavelength waveguiding ability of individual ZnO NRs. Many intriguing phenomena, such as fluorescence intensification at nanorod ends (*FINE*) and the different enhancement degrees at different positions on a single ZnO NR, called a degree of *FINE* (*DoF*), were systematically examined in the process [22,93]. The *FINE* and *DoF* behaviors, as well as their photostability, were characterized with respect to the incident light polarization [1,23,94,95]. It turned out that, unlike the previously discussed FRET, the fluorescence signal-enhancing capability of the ZnO NRs was not limited to a particular distance range between the NRs and bioanalytes [1,28,30,96]. The NRs permitted enhanced fluorescence direction not only from fluorophores directly coupled to the NR surface but also from those placed many tens of nm away from the NR surface, so long as they were within the typical decay length of the evanescent waves of ~100 nm. In more recent research efforts, the mechanical strain dependence of the ZnO NR-guided fluorescence signals was also determined, which will be beneficial for the future integration of ZnO NRs into flexible and wearable biosensors [30,96]. Along with these discoveries, the use of ZnO NRs in fluorescence detection has been extended to determine a range of bioanalytes of DNA, peptides, proteins, and cells [24,26,28].

ZnO NRs were demonstrated for the detection of *Bacillus anthracis* (*B. anthracis*) from a genetically related species of *Bacillus cereus* (*B. cereus*) [24]. An elastomeric polydimethylsiloxane (PDMS) piece holding two reaction chambers was placed over a ZnO NR platform. The ZnO NRs in the two chambers were modified by nonspecifically adsorbing ssDNA probes, one for *B. anthracis* and the other for *B. cereus*. A 6-carboxyfluorescein-conjugated ssDNA fragment of *B. anthracis* was then added to both chambers. Fluorescence signals were detectable only from the chamber that contained the ssDNA probe fully complementary to *B. anthracis*. The study also found that a covalent attachment of the ssDNA probe to the ZnO NRs resulted in higher fluorescence signals relative to the noncovalent, nonspecific adsorption method. The surface hydroxyl group of the ZnO NRs can be conveniently exploited to covalently attach the ssDNA probe via its amine-modified 3′ end to the epoxy group of a silane-treated NR surface. The utility of the ZnO NRs in fluorescence detection was extended to multilayer protein reactions, as displayed in Figure 3E,F [26]. Discernable fluorescence signals were detected only from the NR platforms sequentially treated with immunoglobulin G (IgG) and FITC–anti-IgG, whereas no observable fluorescence was monitored from the NR platforms that underwent sequential treatment with fibronectin (Fn) and FITC–anti-IgG. Similarly, a strong emission was detected from the ZnO NR platform that underwent reactions with biotinylated bovine serum albumin (BBSA) and dichlorotriazinylamino fluorescein-conjugated streptavidin (DTAF–streptavidin). The control NR platform treated with IgG and then DTAF–streptavidin yielded no measurable fluorescence. It was also revealed that enhanced fluorescence detection was possible on ZnO NRs regardless of the different fluorophores, such as FITC, DTAF, cyanine 3 (Cy3), and tetramethyl rhodamine isothiocyanate (TRITC) [26]. These results indicated that ZnO NRs provided a stable reaction environment for biomolecules, which were able to maintain their biological activities on the NR surface after being immobilized noncovalently or covalently. These experimental findings also suggested that ZnO NRs exhibited good chemical stability during the multistep bioreaction processes and high compatibility in signal enhancement with a variety of fluorophores commonly used in bioassays. Beyond the bioanalytes of purified DNA and proteins, the fluorescence-enhancing capability of ZnO NRs was further examined in more complex biological reactions. A ZnO NR platform was employed in a telomeric repeat elongation (TRE) assay with an oligonucleotide specifically recognized by telomerase in HeLa cells [28]. The oligonucleotide was first covalently attached to silanized ZnO NRs, after which a deoxyribonucleotide triphosphate (dNTP) mixture of dCTP, dGTP, dTTP, and biotinylated dATP was added. The dNTPs allowed telomerases in HeLa cell lysates to extend the 3′ end of the oligonucleotide with the telomeric repeat unit of TTAGGG. The biotinylated dATPs in the elongated portion of the oligonucleotide were then reacted with DTAF–streptavidin. The successful extension of the oligonucleotide sequence by telomerase, in turn, led to fluorescence. These results show that, beyond the purified bioanalytes and simple bioassays, even those weak fluorescence signals from the bioanalytes in physiological samples and in complex bioreaction environments could be determined by ZnO NRs. This possibility was later realized in many research efforts, discussed below.

**Figure 4:**
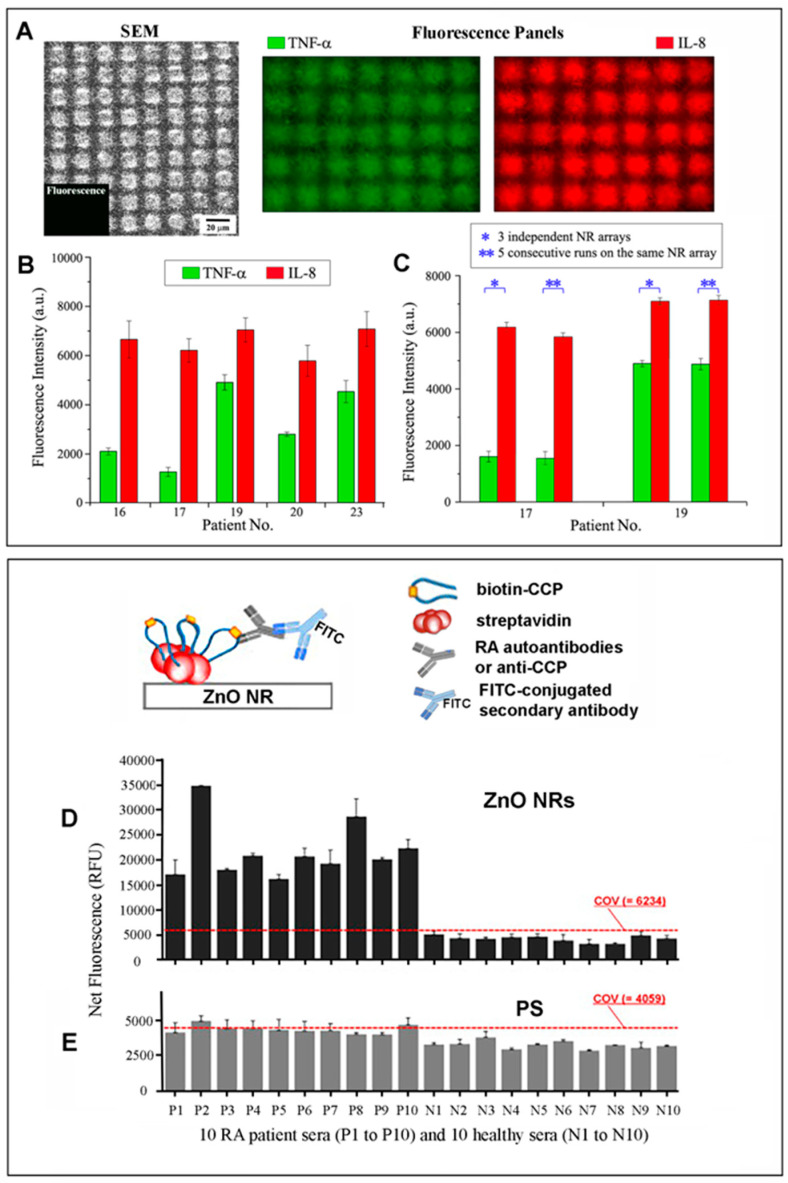
Examples of ZnO NRs used as a signal-enhancing platform for bioanalyte fluorescence detection are displayed. The bioanalytes assayed in (**A**–**C**) are the cytokines TNF-α and IL-8 in AKI patient urine samples, and those in (**D**,**E**) are the RA autoantibodies in RA patient sera. (**A**) The SEM panel displays a top-down view of vertically oriented ZnO NRs grown into a square array where the as-synthesized NRs were free from fluorescence emission, as evidenced in the inset. For the simultaneous detection of TNF-α and IL-8, sandwich-type immunoassays were performed on the ZnO NRs. The fluorescence panels of green and red were obtained from the ZnO NRs, where the two colored panels correspond to the detection channels of TNF-α and IL-8, respectively, due to their respective Alexa 488-labeled and Alexa 546-labeled secondary antibodies. (**B**) The bar graphs display fluorescence readings obtained for both biomarkers from selected patient samples. The fluorescence signals from ∼550 NR square patches per sample were analyzed to obtain the average fluorescence intensity, as well as the associated error bars reported for each patient sample. (**C**) The data show the results of interassay (data shown under *) and intra-assay (data shown under **) variability for the same patients that were carried out on three different ZnO NR arrays and five times on the same platform, respectively. (**A**–**C**) Reproduced with permission from Ref. [29], copyright (2016) Royal Society of Chemistry. (**D**,**E**) The bar graphs summarize the diagnostic assay results of 10 RA patients and 10 healthy sera using the immunodetection scheme shown in the cartoon. The assays were performed on the platform of (**D**) ZnO NRs and (**E**) PS. The red lines inserted in the bar graphs represent the cut-off values (COVs), which were determined from the signals of the negative control samples, i.e., average plus 3 times standard deviation, measured on each platform. On the platform of ZnO NRs, all patient sera showed strong positive signals far above the COV, whereas only a couple of patient sera yielded positive signals that were barely above the COV in the assays using the conventional 96-well PS plate. The net fluorescence measured from each sample was much higher on the ZnO NRs relative to those on the PS. (**D**,**E**) Adapted with permission from Ref. [31], copyright (2011) Elsevier B.V.

One-dimensional ZnO nanomaterials have been used to detect a wide variety of protein analytes, which have included IgG, glucose, anthrax protective antigen 83 kDa (PA83), carcinoembryonic antigen (CEA), alpha-fetoprotein (AFP), prostate-specific antigen (PSA), interleukin-8 (IL-8), tumor necrosis factor-α (TNF-α), anti-cyclic citrullinated peptide (anti-CCP), rheumatoid arthritis (RA) autoantibodies, adenosine triphosphate (ATP), fatty acid-binding protein (FABP), cardiac troponin I (cTnI), myoglobin (Mb), and ketoprofen (KP) [11,29,31,32,33,35,36,86,87,88,97,98,99,100,101,102,103,104,105,106,107]. Two examples of such applications of ZnO NRs as signal-enhancing platforms are shown for the detection of acute kidney injury (AKI) biomarkers and RA autoantibodies in Figure 4. A square array of ZnO NRs was used for the detection of IL-8 and TNF-α, which are cytokine biomarkers implicated in AKI [29]. The typical level of IL-8 in human samples is high enough for detection with a commercially available detection kit. TNF-α, however, is a low-level AKI biomarker whose concentration cannot be readily determined by existing means. ZnO NRs were successfully used in the study to simultaneously detect the concentrations of IL-8 and TNF-α in urine samples obtained from healthy subjects, as well as intensive care unit (ICU) patients. A sandwich-type immunoassay performed on the ZnO NR array involved a series of reaction steps, starting with a mixture of unlabeled, primary anti-IL-8 and anti-TNF-α antibodies, followed by BSA passivation. Then, their reactions with a mixture of the two cytokines and, lastly, with a mixture of fluorophore-conjugated secondary antibodies took place. For multiplexed detection, the secondary antibodies were labeled with Alexa488 for TNF-α and Alexa546 for IL-8. Samples from well over 50 patients were analyzed accordingly on a square array platform of vertically oriented ZnO NRs. Figure 4A displays representative fluorescence images from the multiplexed detection of TNF-α and IL-8 carried out on the ZnO NRs. The bar graphs in Figure 4B,C show fluorescence intensities measured for the two cytokines from different patients as well as the intra- and inter-assay variability results of the ZnO NR-based detection. The LODs of the cytokine immunoassays carried out on the ZnO NR platform were determined to be 5.5 fg/mL for IL-8 and 4.2 fg/mL for TNF-α. The detection sensitivity was much better when compared with the LODs of 7.5 pg/mL for IL-8 and 5.5 pg/mL for TNF-α obtained by a conventional ELISA-based kit. The possibility of the ZnO NR array for use in clinical diagnosis was further assessed by first quantifying the TNF-α concentrations in samples from patients at varying AKI stages. The results were then used to predict TNF-α levels expected from those individuals belonging to the groups of no AKI (healthy subjects), AKI developed within 24 h of ICU admission, and AKI developed after the first 24 h of ICU admission. ZnO NR-based diagnostic assays were also carried out in another investigation involving RA patient sera [31]. The levels of anti-CCP RA autoantibodies in patients, as well as healthy subjects, were determined by fluorescence measurements. The assay results obtained on ZnO NRs were then compared to those from a polystyrene (PS) platform. The bar graphs in Figure 4D,E summarize the results obtained from the same set of RA patients, as well as healthy subject samples. The study concluded that the ZnO NR platforms, even in assays involving human sera, could resolve very low fluorescence signals from bioanalytes that could not be determined on a conventional PS platform. In addition, ZnO NRs promoted enhanced detection by aiding the control of the CCP orientation. By introducing a biotinylated CCP to a streptavidin-functionalized ZnO surface, the RA autoantibody-specific motif around the citrulline of CCP could be better exposed for tight binding with anti-CCP.

**Figure 5:**
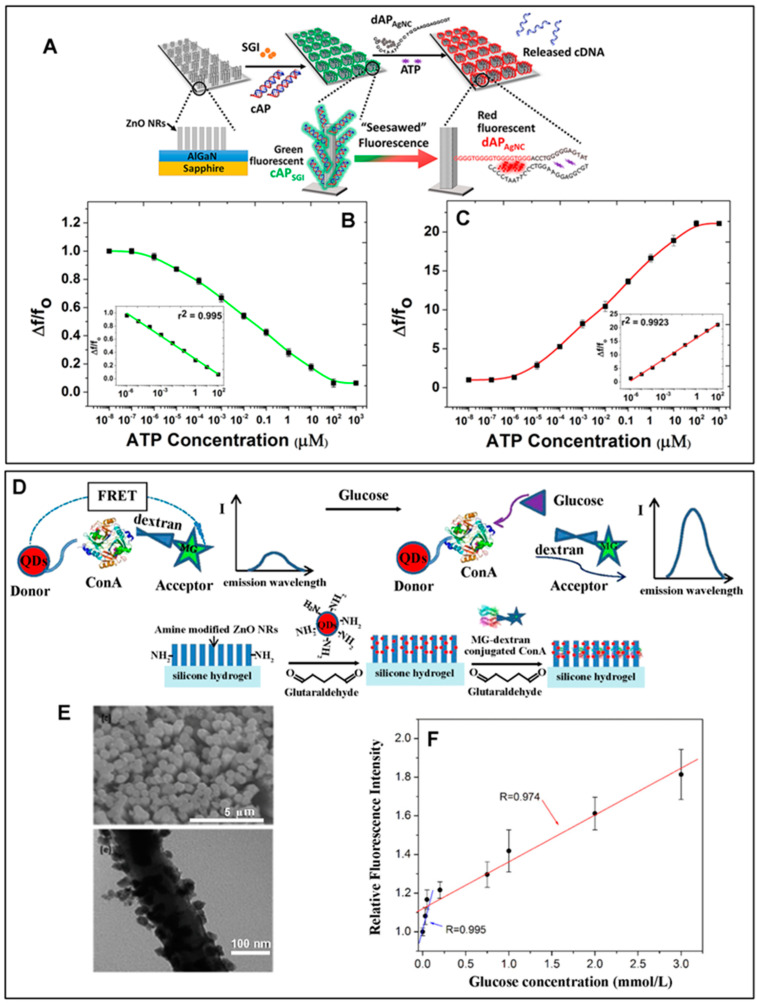
More examples of ZnO NRs used in fluorescence-based biodetection are presented. (**A**) The schematic illustration outlines the ATP detection scheme based on the green and red fluorescence signals associated with two split aptamers—a capture aptamer intercalated with green-fluorescent SGI (cAP_SGI_) and a detection aptamer with an extended DNA sequence required for the formation of red fluorescent AgNCs (dAP_AgNC_)—while employing vertical ZnO NRs as a signal-enhancing platform. The addition of ATP resulted in a decrease in green fluorescence from cAP_SGI_ and a multi-fold increase in red fluorescence from dAP_AgNC_. (**B**,**C**) The ATP concentration-dependent fluorescence intensity changes, Δf/fo, are plotted for (**B**) SGI and (**C**) AgNCs. The insets display the magnified views of the linear regimes. (**A**–**C**) Adapted with permission from Ref. [32], copyright (2016) Elsevier B.V. (**D**) The schematic illustration depicts the glucose detection scheme of a FRET transducer made of Con A-conjugating CdSe/ZnS QDs as a donor and MG as an acceptor while employing a platform of ZnO NRs for signal enhancement. (**E**) The SEM micrographs in the top and bottom panels show a top-down view of the ZnO NRs deposited on a silicone hydrogel and a magnified view of a ZnO NR coated with CdSe/ZnS QDs. (**F**) The plot displays the linear relationship between the measured fluorescence intensity and the concentration of glucose. (**D**–**F**) Adapted with permission from Ref. [11], copyright (2016) Elsevier B.V.

Vertical ZnO NRs fabricated on a sapphire substrate were employed for the detection of ATP by monitoring fluorescence switching behaviors [32]. Figure 5A outlines the overall approach used for ATP detection. A split ATP-binding aptamer (capture aptamer) was hybridized with its complementary DNA sequence (cDNA) and intercalated with SYBR green-I (cAP_SGI_). The amine-derivatized cAP_SGI_ was then covalently attached to APTES-modified ZnO NRs. Another split ATP-binding aptamer with an extended DNA sequence (detection aptamer) was linked with Ag nanoclusters (dAP_AgNC_). In the as-prepared constructs, the red fluorescence of dAP_AgNC_ was negligible compared to the green emission of cAP_SGI_. The higher cAP_SGI_ signal was due to the fluorescence-enhancing capability of the ZnO NRs onto which cAP_SGI_ was covalently attached. The addition of ATP decreased the green fluorescence from cAP_SGI_ and increased the red fluorescence from dAP_AgNC_. This was because the two split aptamers were able to form a cAP_SGI_-ATP-dAP_AgNC_ complex by binding with ATP, reducing the distance between dAP_AgNC_ and ZnO NRs. At the same time, the addition of ATP induced the release of the cDNA from cAP_SGI_. No longer bound to the signal-enhancing ZnO NRs, the SGI release led to a reduction in green fluorescence. Figure 5B,C display the intensity changes associated with the green and red fluorescence from SGI and AgNC, respectively, as a function of ATP concentration. This fluorescence switching behavior of the ZnO NR sensor showed a linear relationship in the ATP concentration range of 1 pM–100 µM with a LOD of 1 pM. A different strategy for using 1D ZnO nanomaterials in fluorescence detection involved magnetic beads conjugated with hydrothermally grown ZnO NRs (MB-ZnO NRs) [33]. MB-ZnO NRs were then utilized in a microfluidic chip for enhanced apta-sensing of cTnI. A dsDNA probe was first constructed from a cTnI aptamer after hybridization with its cDNA and intercalation with SGI. The probe was then immobilized onto silanized MB-ZnO NRs. The MB-ZnO NRs with the dsDNA probe were integrated into a microfluidic chip containing a low reflective matrix of graphite-PDMS. The microfluidic device with serpentine channels had different zones for probe–target incubation and fluorescence image acquisition, while a magnetic field enabled the flow of the MB-ZnO NRs to the different areas of the microfluidic device. The binding of cTnI to the cTnI aptamer resulted in the dehybridization of cDNA from the aptamer and the subsequent release of SGI. Thus, the decreased amount of SGI on the signal-enhancing ZnO NRs resulted in reduced green fluorescence. A LOD of 252.4 pg/mL for cTnI was achieved by the MB-ZnO NR scheme.

In another study, ZnO NRs were utilized for CEA detection after determining the NR diameter that produced the largest fluorescence enhancement for a model fluorophore of R6G [35]. ZnO NRs with diameters of ~150, 180, 230, 270, 320, and 410 nm were grown on Au-coated Si substrates. To control the diameters, a PMMA mask layer was coated atop the ZnO seed layer on the substrate, after which the different sizes were etched by using electron beam lithography for the guided hydrothermal growth of the NRs. Both the experimental evidence on the R6G emission and finite difference time domain (FDTD) simulations revealed that the fluorescence enhancement arose from high-order waveguide modes in ZnO NRs, where the modes were dependent on the NR diameter, substrate, and the refractive index of the surrounding medium. A sandwich CEA immunoassay using Cy3-labeled anti-IgG was then built on the ZnO NR platform for fluorescence measurements. When using the maximum fluorescence-enhancing ZnO NR platform of 270 nm in diameter, a LOD of 10 fg/mL was achieved from the CEA detection.

A ZnO NR array with an NR diameter of ~120 nm was also employed as a signal-enhancing platform to promote the detection of the FRET signal in an anti-FRET glucose sensor [11]. The overall sensing strategy for glucose detection is illustrated in Figure 5D. The FRET donor–acceptor pair used in the study was CdSe/ZnS QDs and dextran-bound malachite green (MG–dextran). To conjugate the FRET pair to the ZnO NR array, a 150 µm thick ZnO NR layer was prepared on a polysiloxane hydrogel. CdSe/ZnS QDs were then linked to the ZnO NRs. Figure 5E shows the ZnO NRs, as well as NR-coated CdSe/ZnS QDs in the top and bottom SEM panels, respectively. MG–dextran with concavalin A (Con A), an enzyme with a specific affinity for glucose, was attached to the QDs in the next step. Initially, the proximity of the donor and acceptor compounds led to quenched fluorescence from the QDs. In the presence of glucose, the interaction between Con A and dextran became outcompeted by the strong affinity of Con A to glucose. This caused the release of MG–dextran from Con A, as well as from the QDs, which restored the QD fluorescence at 652 nm. A linear response between the restored fluorescence intensity and glucose concentration was observed for the span of 0.03–3 mM, as displayed in Figure 5F. Fluorescence images of the patterned FRET sensor on the silicone hydrogel were also obtained, where the pixel intensities in the images were found to increase linearly in the glucose concentration range of 0.03–0.6 mM. Another study employed ZnO NRs as a FRET acceptor for the detection of CEA [36]. Vertical ZnO NR arrays with an NR diameter of ~30 nm and length of ~2–3 µm were grown hydrothermally on a glass substrate, after which the NRs were silanized for the subsequent reactions with CEA antibodies labeled with the FRET donor of CdSe/ZnS QDs. On bare ZnO NRs, the measured QD fluorescence at 605 nm was weak due to the FRET between the QD donor and the ZnO NR acceptor. ZnO NRs were further modified with anti-FRET layers of biotin–streptavidin complexes to increase the separation distance from the QDs and to reduce the energy transfer between the NRs and QDs. This modification led to increased QD fluorescence signals, which enabled a wide detection range of 0.001–100 ng/mL and a LOD of 0.001 ng/mL for CEA.

##### 2.2.4.2. Single ZnO and ZnO-Related Nanorods in Fluorescence-Based Biodetection

In addition to the applications of an ensemble of 1D ZnO nanomaterials discussed so far, the potential use of single NRs in optical biodetection has been assessed [90,108,109,110,111,112,113,114]. Although the nanophotonic applications of individual ZnO NRs and related 1D nanomaterials have been tested for the delivery, collection, and even separation of the different components of the optical signals, their practical utilities in biodetection have remained unexplored [92,115]. For this undertaking, fluorophore-coupled bioanalytes attached to the surface of single ZnO NRs were examined in a series of studies, and the research efforts revealed interesting phenomena, such as fluorescence intensification, spatial localization, and the temporal persistence of the bioanalyte signal [1,22,23,30,93,94,96]. The model bioanalytes in the single ZnO NR studies were later extended to include not only fluorophores or fluorophore-coupled proteins distributed to all crystalline facets of the NR but also fluorophore-coupled peptide fragments designed to recognize and bind only to a specific NR facet [1,22,23,30,93,94,96].

**Figure 6:**
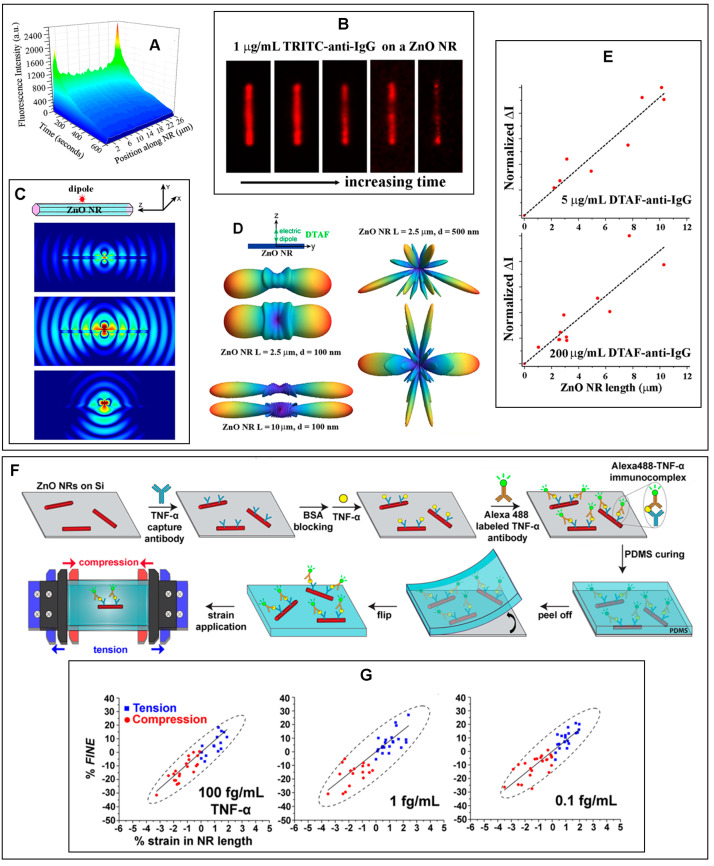
Exemplary properties of individual ZnO NRs and their utilities in biodetection are shown. (**A**) The contour map displays the fluorescence intensity of 200 μg/mL DTAF-anti-IgG deposited on a 25 μm long ZnO NR. The DTAF-anti-IgG fluorescence signals were intense on the NR end relative to the NR side facets. The fluorescence signals on the single ZnO NR also persisted under constant irradiation, reaching a half-life (t_1/2_, the time point at which there is a 50% reduction of the intensity measured at t = 0) of ~1 min. They were extended much beyond those measured on the conventional substrates of PMMA and PS, whose t_1/2_ were ~15 and 30 s, respectively. Reprinted with permission from Ref. [22], copyright (2014) Royal Society of Chemistry. (**B**) The time-lapse fluorescence panels display the spatial and temporal emission behaviors of 1 μg/mL TRITC-anti-IgG deposited on a ZnO NR. (**C**) The FDTD simulation results in the top, middle, and bottom panels correspond to the radiation patterns from a single 576 nm emitter whose polarization is along the X, Y, and Z directions, respectively. (**D**) The nanomaterial size effect of a ZnO NR on *FINE* was evaluated by FDTD simulations. Far-field radiation patterns were obtained from a 517 nm electric dipole and shown for each ZnO NR of the specified length (L) and width (d). The spatial radiation patterns observed from the Z and X directions are displayed in the top and bottom simulation panels, respectively, for the ZnO NRs with the specified dimensions. (**E**) The plots show the effect of the NR length on *FINE* and *DoF*. Different concentrations of DTAF-anti-IgG were coupled to ZnO NRs of various lengths, and the DTAF-anti-IgG fluorescence signals on the ZnO NRs were measured. In all cases, the normalized fluorescence intensity value of ΔI (I_avg,NRef_ − I_avg,NRsf_) indicated that the *DoF* increased as the NR length became longer. The subscripts of avg, NRef, and NRsf stand for average, NR end facets, and NR side facets, respectively. (**B**–**E**) Reprinted with permission from Ref. [23], copyright (2015) Royal Society of Chemistry. (**F**) The scheme illustrates the overall fabrication process of the TNF-α sandwich immunoassay based on individual ZnO NRs. The NR immunoassay platform was integrated into a PDMS elastomer for the application of uniaxial tensile (blue) and compressive (red) strain with a microvice during fluorescence measurements. (**G**) The plots display % *FINE* versus % strain in NR length for TNF-α concentrations of 100, 1, and 0.1 fg/mL. Squared data points in blue correspond to the NRs undergoing tension, while circled data points in red refer to the NRs undergoing compression. The solid black lines represent the linear fits for the data points, while the black dashes show the 95% confidence ellipses. Positive values on the *x*-axis of % strain in NR length denote tension, while negative values indicate compression. (**F**,**G**) Reprinted with permission from Ref. [30], copyright (2024) MDPI.

*FINE* and *DoF* were first discovered and characterized by examining the fluorescence emission from DTAF-conjugated anti-IgG on individual ZnO NRs [22]. Extensive experimental and FDTD simulation efforts followed to elucidate key nanomaterial as well as biomolecular factors that affect *FINE* and *DoF* [23]. *DoF* serves as a measure of the signal intensification at the NR ends relative to the NR main body. Figure 6 presents the fluorescence-enhancement properties of individual ZnO NRs that were determined experimentally and through computer simulations. The fluorescence from DTAF-anti-IgG, as well as TRITC-anti-IgG, showed an NR position-dependent profile, in which its signal intensity was revealed to be highly localized and significantly intensified at the NR ends relative to the NR main body. This is shown in the 3D contour plot and the time-lapse fluorescence panels in Figure 6A,B. The fluorescence intensity and photostability measured on the NR were both higher than those on the conventional substrate of PS or PMMA, even though more biomolecules tended to adsorb on the polymers relative to ZnO. *FINE* and *DoF* were affected by factors such as the NR length/width, NR orientation with respect to the substrate, protein concentration, and the absorption/emission wavelengths of the fluorophore. These aspects can be evidenced from the results provided in Figure 6C–E. In particular, *DoF* was found to be highly dependent on the NR length. The longer the NR was, the greater the fluorescence signal enhancement occurred, and the better the signal was directed along the NR main body towards the NR ends, as seen in Figure 6D,E. In addition, vertically oriented NRs with respect to the growth substrate were shown to exhibit a larger *DoF* than laterally oriented NRs. The vertical configuration permitted a minimal loss of the NR-guided fluorescence signal to the underlying substrate and, at the same time, a better alignment between the detector and the NR ends from which the guided fluorescence signals radiated out to the far field. As for the bioanalytes, higher protein concentrations resulted in greater *DoF*s since a larger number of biomolecular signals could be coupled and guided by the NR. FDTD simulations provided insight into the spectroscopic profiles of the fluorophores to *FINE* and *DoF*. Fluorophores with shorter emission wavelengths showed a slightly better directional coupling along the NRs. These findings will be important for the optimization of individual ZnO NRs to maximize *FINE* and *DoF* in fluorescence-based biodetection.

In ensuing research efforts, it was further shown that mechanical strain can affect the subwavelength waveguiding behavior of ZnO NRs [30,96]. The fluorescence intensity of R6G measured on ZnO NRs was found to increase and decrease upon the application of tensile and compressive strain, respectively [96]. These trends in the strain-induced changes in fluorescence intensity persisted even though the fluorescence source was separated from the NR surface by tens of nm [30]. This was shown in a study to detect TNF-α through the formation of Alexa488–TNF-α immunocomplexes on ZnO NRs after a series of sandwich-type immunoreactions, as schematically shown in Figure 6F [30]. Both *FINE* and *DoF* were found to be linearly dependent on the types and amounts of strain, regardless of the protein concentrations. These effects can be seen in Figure 6G. It was also determined that the NR length played a critical role in producing a large response in the fluorescence intensity with respect to the applied strain, as well as protein concentration. The longer the NR was, the greater the changes in both *FINE* and *DoF* that were induced for a given amount of strain applied. In addition, longer NRs relative to shorter ones permitted a stronger correlation between the protein concentration and fluorescence intensity. Hence, the NR length and the amount of strain applied to ZnO NRs can be synergistically optimized for highly sensitive detection of biomolecules at ultra-trace levels, even in a wearable or flexible device setting.

Other transparent conducting oxide (TCO) NRs with physical dimensions and optical properties similar to those of ZnO NRs have also been demonstrated for guiding and collecting optical signals [92,115]. For example, a 1D SnO_2_ NR fixed on the tapered tip of an optical fiber was exploited as a biocompatible waveguide for single-cell endoscopy [115]. The NR-based endoscopy probe was connected to an excitation laser and a spectrometer. The NR was capable of delivering a payload of QDs into the intended intracellular compartments of a living cell following the NR probe insertion into the cell and ~1 min of UV illumination. For this, a cargo of commercially available QDs (QD655) was attached to the NR tip through a photoactivated crosslinker of sulfosuccinimidyl 6-(3′-(2-pyridyldithio)-propionamido) hexanoate, which could be cleaved by low-power UV radiation. The thin diameter and uniform geometry of the NR made it less invasive to the cell than directly inserting the optical fiber tip. This work marked the first demonstration of individual NR waveguides in single-cell endoscopy, exemplifying their practical usefulness in high-resolution subcellular imaging and fluorescence tracking.

#### 2.2.5. Applications of 2D ZnO Nanomaterials in Fluorescence Biosensors

Although less common than 0D or 1D forms, 2D ZnO nanomaterials have been demonstrated for their use in the fluorescence detection of bioanalytes, such as amyloid-beta 42 (Aβ_42_), soluble epidermal growth factor receptor (sEGFR), and green fluorescent protein (GFP) [37,38,116]. For instance, a plasmonic chip of ZnO/Ag/PMMA was constructed by coating a Ag layer on a PMMA substrate followed by the magnetron sputtering of a ZnO thin film [37]. The ZnO/Ag/PMMA plasmonic chip was then employed to detect GFP. For the fluorescence detection of Cy5-labeled GFP, a bispecific antibody with affinity for both GFP and ZnO surfaces was adsorbed on the plasmonic chip. Due to its high affinity towards ZnO, the bispecific antibody was able to form a dense layer on the ZnO thin film. This allowed a larger amount of antibody molecules to react with Cy5-labeled GFP, and stronger fluorescence signals were obtained from the sensor relative to the case without a ZnO thin film. Fluorescence signals from the Cy5-labeled GFP were further improved by the surface plasmon resonance (SPR) property of Ag and better coupling with the incident light by a periodic grating engineered in the Ag layer. The use of the ZnO/Ag/PMMA chip in the GFP detection was effective for a concentration range of 10 pM–100 nM with a LOD of 7 pM. The same ZnO/Ag/PMMA plasmonic chip was also used for detecting a tumor marker, sEGFR [38]. The ZnO-based plasmonic chip rendered a wide sEGFR detection range of 700 fM–10 nM with a LOD of 700 fM.

### 2.3. Photoluminescence: Near-Band-Edge Emission and Deep-Level Emission

#### 2.3.1. Contributions of ZnO Nanomaterials in Photoluminescence-Based Biodetection

The inherent photoluminescence (PL) of ZnO nanomaterials can be controlled by their well-established synthesis methods. The gas-phase approaches for ZnO nanomaterials relying on the vapor–liquid–solid (VLS) growth or vapor–solid (VS) growth mechanisms are typically carried out in a CVD or metal organic CVD (MOCVD) chamber [1,117,118]. ZnO nanomaterials grown via gas-phase reactions are known to exhibit only a very sharp, near-band-edge (NBE) emission associated with the bandgap of ZnO. Emissions in the visible range are absent due to negligible surface or crystalline defects [1,119]. When such high-quality ZnO nanomaterial is excited above its bandgap energy of ~3.37 eV, an intense NBE emission dominantly occurs at ~388 nm due to excitonic transitions [120,121,122]. ZnO nanomaterials can also be synthesized by solution-phase procedures, such as hydrothermal, sol-gel, and co-precipitation methods. As-synthesized ZnO nanomaterials produced in solution-phase reactions typically show considerable visible and near IR emissions in addition to their NBE in the UV range [1]. Additionally, ZnO nanomaterials can be prepared to exhibit visible emissions by extrinsically modifying their surface with transition metal ions, nitrogen-doping, or dye sensitizers. Intrinsic or extrinsic in origin, ZnO nanomaterials containing surface defects, chemical impurities, or physical deformations produce broad emissions in the visible and near IR regions that are collectively referred to as deep-level emissions (DLEs) [122,123,124]. The peak broadness of DLEs may be due to simultaneous emissions from many different deep levels corresponding to red, yellow, green, and blue emissions. Varying explanations for these emissions are found in the literature, sometimes with differing opinions. For instance, a green DLE band at 495–515 nm has been attributed to the defects of oxygen vacancies, Zn vacancies, or interstitial Zn [123,124,125,126]. A yellow DLE band centered at 527 nm is commonly attributed to the presence of interstitial oxygen sites, which are involved in the electron transitions from the conduction band (CB) to the deeply trapped hole in the single negatively charged interstitial oxygen ion [127].

PL signals are known to sensitively respond to small changes occurring at a material’s interface [128,129]. In addition, nanomaterials present a high surface-to-volume ratio relative to their bulk counterparts. This further enables a more sensitive detection when a biorecognition event at the interface of a bioreceptor-conjugated nanomaterial is monitored for any changes in its PL signals in a label-free manner. As such, the NBE and DLE peaks of ZnO nanomaterials have been evaluated for change in intensity, a shift in position, the appearance of a new band, and the disappearance of an existing band, each of which can serve as a signal-transducing mechanism in biodetection. This section introduces those biodetection works focused on the intrinsic ZnO PL of NBE and DLE rather than extrinsically modified PL signals.

#### 2.3.2. Applications of 0D ZnO Nanomaterials in Photoluminescence Biosensors

Various bioanalytes, such as glucose, urea, trypsin, ciprofloxacin, N-acyl homoserine lactone (AHL), and calf thymus (CT) DNA, have been detected by monitoring the NBE and/or DLE peaks of ZnO NPs [9,39,40,41,130,131]. In a study exploiting PL quenching, ZnO NPs were used as an enzymatic sensor to detect glucose [9]. The production of H_2_O_2_ from the oxidation of glucose triggered quenching of the ZnO NPs’ PL, both for the NBE emission at 374 nm and the DLE at 525 nm. The decreased PL intensities were proportional to the glucose concentration, leading to a linear range of 30–130 mM and a LOD of 10 mM. In a different study relying on PL enhancement instead, cysteamine-functionalized ZnO NPs (Cys-ZnO NPs) were fabricated for the detection of AHL [39]. AHL is a quorum-sensing signaling molecule of Gram-negative bacteria. With increasing AHL concentration, the PL intensity of Cys-ZnO NPs at 468 nm also intensified due to the interaction between the carbonyl group of AHL and the amine group of Cys. The linear detection range of the Cys-ZnO NP biosensor was 10–120 nM in artificial urine media (AUM). In another research effort, a newly generated DLE of ZnO NPs with self-assembled diphenylalanine (FF) was applied for the detection of trypsin, a serine protease [40]. In the presence of trypsin, a weakened interaction between the FF and ZnO produced a new DLE of ZnO at 550 nm. The DLE intensity increased with trypsin concentration, resulting in a linear range of 0–160 ng/mL and a LOD of 0.1 ng/mL. A different strategy of monitoring the shift in the PL peak was also demonstrated in a study to detect CT DNA by a nanocomposite probe of Au-ZnO NPs [41]. The PL peak of the Au-ZnO NPs at 520 nm showed an appreciable blue shift in the presence of CT DNA relative to the control DNA strands of *Escherichia coli* (EC) and *Micrococcus lysodeikticus* (ML). The CT DNA detection yielded a linear range of 0.1–0.7 µM with a LOD of 36 nM.

#### 2.3.3. Applications of 1D ZnO Nanomaterials in Photoluminescence Biosensors

**Figure 7:**
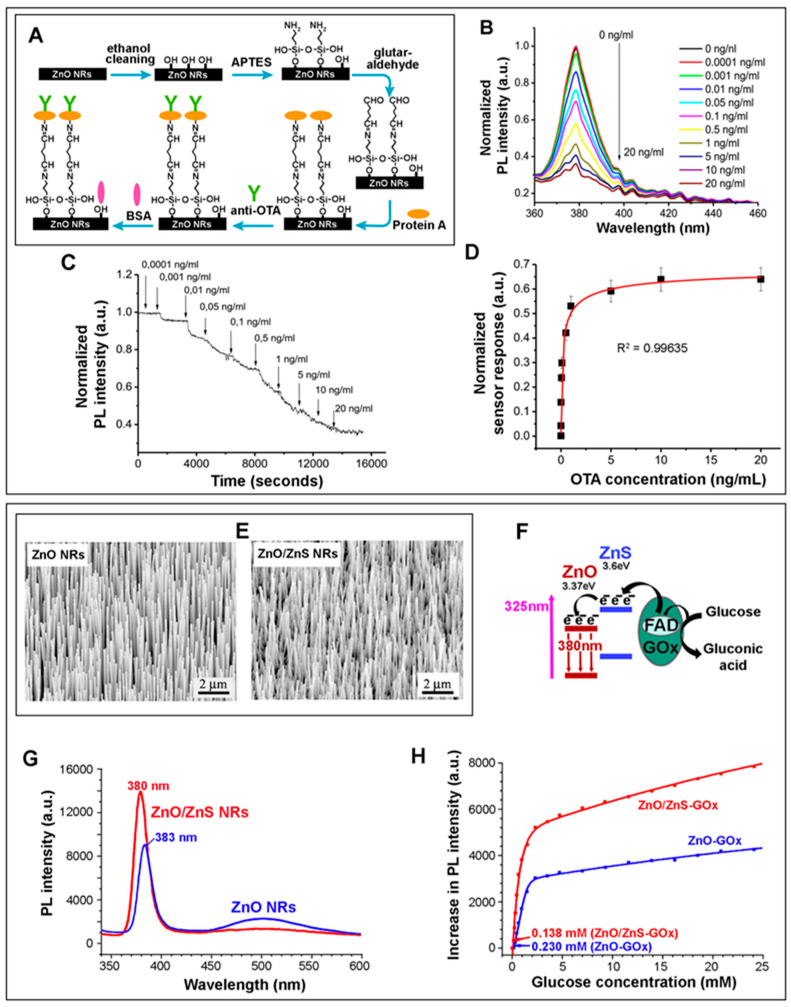
The presented examples of biodetection exploit the PL of ZnO nanomaterials as a signal transduction means, where the changes in their emissions, such as NBE and DLE, are monitored to quantify the bioanalytes of OTA and glucose. (**A**) The schematic displays the fabrication processes for an OTA detection platform using ZnO NRs. The immunodetection layers of Protein A and anti-OTA were attained on the ZnO NRs through a series of reactions such as silanization, introduction of amino groups by treatment with APTES, introduction of aldehyde groups by modification with glutaraldehyde, covalent immobilization of Protein A, complexation with anti-OTA, and finally, BSA blocking. (**B**) The graph shows the PL spectra from steady-state conditions after the sensor platform was introduced with OTA of varying concentrations up to 20 ng/mL. (**C**) The plot displays the change in the normalized PL intensity measured at 379 nm when different concentrations of OTA were added over time. (**D**) The plot provides the ZnO NR-based immunosensor response to different OTA concentrations. The normalized sensor response plotted was determined by subtracting the normalized intensity from 1. (**A**–**D**) Adapted with permission from Ref. [43], copyright (2017) Elsevier B.V. (**E**) The SEM micrographs show the ZnO NRs and ZnO/ZnS core/shell NRs in the left and right panels, respectively. (**F**) The diagram depicts the electron injection from the flavine moiety to the ZnO/ZnS NRs and the subsequent increase in the UV emission from the ZnO/ZnS-MAA-GOx bioconjugates. (**G**) Upon modification of the NR surfaces with MAA-GOx, the PL spectra were obtained from the platforms of ZnO NRs (blue) and ZnO/ZnS NRs (red). (**H**) The plot displays the increased PL intensity versus glucose concentration for ZnO-MAA-GOx (blue) and ZnO/ZnS-MAA-GOx (red). The glucose LODs determined for ZnO-MAA-GOx and ZnO/ZnS-MAA-GOx were 0.23 and 0.14 mM, respectively. (**E**–**H**) Adapted with permission from Ref. [13], copyright (2011) Elsevier B.V.

1D ZnO nanomaterials have also been applied for both the solution- and solid-state PL detection of glucose, lactate, cholesterol, ochratoxin A (OTA), grapevine virus A-type (GVA) proteins, and EC DNA [12,13,42,43,44,132,133,134]. For instance, ZnO NRs with NBE emission at 387 nm and DLE at 575 nm were used for EC DNA detection [42]. Successive addition of EC DNA led to a decrease in the DLE and, at the same time, the formation of a new peak at ~426 nm, potentially due to hydrogen bonding between the ZnO NRs and the DNA. The DLE quenching and the formation of the 426 nm peak took place under equilibrium, which was indicated by a near isosbestic point observed at ~517 nm. This process generated white light from the ZnO NRs, whose signals were selective to EC dsDNA over the control samples of CT, ML, and EC ssDNA. Using this scheme, the EC DNA detection by ZnO NRs resulted in an operational range of 0.102–0.894 µM and a LOD of 28.4 nM. Another effort involved the development of an immunosensor for ochratoxin A (OTA) based on Protein A-modified ZnO NRs on a glass substrate [43]. The ZnO NR-based sensor fabrication and detection results are displayed in Figure 7A–D. The synthesized ZnO NRs with NR dimensions of ~90 nm in diameter and ~470 nm in length exhibited the characteristic NBE at ~379 nm and a relatively weak, broad DLE at ~540 nm. Protein A was covalently attached to pre-silanized ZnO NRs for the oriented binding of the anti-OTA antibodies (anti-OTA) and to enhance the NBE intensity via dipole–dipole and electrostatic interactions. The binding of OTA to anti-OTA altered the electrostatic interactions between Protein A/anti-OTA and ZnO NRs, potentially inducing partial dissociation and degradation of the Protein A/anti-OTA immunocomplexes. The NBE intensity decreased with increasing OTA. The OTA detection rendered an operational concentration range of 0.1–1 ng/mL with a LOD of 0.01 ng/mL.

ZnO NRs hydrothermally grown to 150–250 nm in diameter were also used in a PL-based glucose detection after coupling the NRs with Au NPs of 5–10 nm in size [12]. The NRs without sputtered Au NPs exhibited an NBE at 382 nm and a DLE at 575 nm. Upon coating the NR surface with sputtered Au NPs, excited electrons in Au could be readily transferred to the ZnO CB, resulting in greater radiative recombination for NBE and suppressed DLE. The subsequent detection of glucose was carried out by monitoring the NBE intensity change due to the photooxidation of glucose by ZnO under UV illumination. The photoexcited electrons in the ZnO CB transferred to the LUMO of glucose, producing the superoxide anion of O_2_^−^ after a reaction with O_2_. At the same time, the photogenerated holes in the ZnO valence band (VB) migrated to the glucose’s HOMO, reacting with OH^−^ or H_2_O to produce the hydroxyl radical of •OH. The photogenerated O_2_^−^ and •OH then oxidized glucose, which, in turn, reduced the electrons in the ZnO CB that could undergo radiative recombination. The photooxidation of glucose consequently resulted in the reduction in NBE. The glucose sensor exhibited a linear range of 0.01–2 mM with a LOD of 0.01 mM. In a different work of glucose detection, ZnO/ZnS NRs were used after activating the NR surface with an esterification reaction between mercapto-acetic acid (MAA) and n-hydroxysulfo-succinimide (sulfo-NHS) to covalently couple the MMA-modified NR surface to GOx [13]. In this scheme, the presence of glucose enabled the injection of the electrons from the glucose oxidation into the ZnO CB and their subsequent transition to the VB. Therefore, the addition of glucose led to a greater level of radiative decay and an increased ZnO NBE intensity at 380 nm. The glucose detection results obtained from the platforms of ZnO NRs versus ZnO/ZnS NRs are compared in Figure 7E–H. The emission at ~500 nm from the ZnO-MAA-GOx was attributed to the intrinsic green luminescence arising from the surface defects within the ZnO structure. With the introduction of the ZnS sheath, the NR surface defects were effectively passivated, as the formation of oxygen vacancies and dangling bonds on the ZnO surface was prevented by the presence of the sheath. This, in turn, enhanced the PL signal of the NR biosensor and eliminated the emission at ~500 nm. The ZnO/ZnS-MAA-GOx NR biosensor showed a linearly increasing PL intensity for the glucose concentration range of 3.51–24.1 mM with a LOD of 0.14 mM.

#### 2.3.4. Applications of 2D ZnO Nanomaterials in Photoluminescence Biosensors

As for 2D ZnO thin films, research efforts have been carried out for their use in the PL detection of glucose and GVA proteins [45,135]. In the example of GVA proteins, the adsorption of anti-GVA antibodies to a ZnO thin film led to the appearance of a DLE at 425 nm due to the formation of Zn-S bonds [45]. A subsequent reaction with GVA resulted in a decrease in the DLE intensity due to the weakening Zn-S bonds between ZnO and anti-GVA. The ZnO thin-film sensor was responsive to GVA proteins in the concentration of 1 pg/mL–10 ng/mL.

### 2.4. Chemiluminescence

#### 2.4.1. Overview of Chemiluminescence in Biodetection

Chemiluminescence (CL) is broadly defined as light emission from a chemical reaction. It is generally considered to have a lower sensitivity, narrower dynamic range, greater tendency of signal saturation, and no multiplexing capability compared to the previously discussed fluorescence-based methods. However, the relatively low technical requirements for signal visualization and imaging, similar to those of colorimetry, make them attractive. CL detection can also provide a very low background signal in principle since the luminescence is triggered by only those chemical reactions related to the presence of analytes, as opposed to fluorescence-based techniques, whose excitation conditions can be prone to unwanted emissions from background molecules [136]. CL is often incorporated into bioassay protocols by an enzyme and a chemical substrate. In many cases, 3-aminopthalhydrazide (luminol, LH_2_) is used as a substrate, along with an enzyme of HRP that is conjugated to an antibody to react with a target bioanalyte. HRP catalyzes the oxidation reaction between LH_2_ and an oxidant such as H_2_O_2_, producing an electronically excited 3-aminophthalate intermediate [137,138]. The decay of this intermediate to stable 3-aminophthalate leads to low-level luminescence at 450 nm. Hence, the enzyme amount and the amount of light generated can be correlated with the bioanalyte concentration.

#### 2.4.2. Contributions of ZnO Nanomaterials in Chemiluminescence-Based Biodetection

The main technical challenges of CL-based biodetection are related to the low quantum efficiencies and weak luminescence from CL reactions. To increase the weak CL intensity and to delay signal decay, enhanced chemiluminescence (ECL) is often used instead. Conventional ECL platforms have additional enhancing molecules such as phenol, naphthol, aromatic amine, and benzothiazole. Research advances made in nanomaterials have impacted the CL field and brought significant enhancement of the weak CL emission. For use as excellent CL emitters, nanomaterials should have large molar extinction coefficients, high quantum yields, surface trap-controllable luminescence, low photobleaching, minimal photodegradation, and chemical stability. ZnO nanomaterials meet these requirements, and therefore, 0D and 1D forms of ZnO nanomaterials have emerged as new enhancer molecules in ECL-based biodetection and bioimaging [72,73]. Nanomaterials can participate in CL reactions both directly and indirectly [136]. The former is achieved by directly generating electrons and holes inside the nanomaterials. A redox chemical reaction can induce this by injecting electrons and holes into the nanomaterials [139]. The injected electrons and holes can then facilitate the electron–hole annihilation process that produces CL emission. In the indirect participation of nanomaterials, the presence of a nanomaterial promotes the creation of a radical, whose role in a downstream chemical reaction produces a key intermediate that ultimately leads to CL. Depending on the chemical reactions utilized, nanomaterials can catalyze the formation of •OH, •CO_3_^−^ (carbonate radical), •HCO_4_^−^ (peroxymonocarbonate radical), or •L^−^ (luminol radical) [140,141,142]. For instance, the superoxide anion of O_2_^−^, yielded from the reaction between H_2_O_2_ and the nanomaterial-catalyzed •OH radical, leads to the formation of 1,2-dioxetanedione, which functions as an essential intermediate for establishing CL. ZnO nanomaterials typically participate in CL-based biodetection through the indirect mechanism.

#### 2.4.3. Applications of 0D/1D ZnO Nanomaterials in Chemiluminescence Biosensors

ZnO NPs of ~100 nm in size were demonstrated for the ECL-based detection of CEA [34]. In this work, a sandwich-type ELISA assay was developed by exploiting ZnO NP-enhanced CL from the reaction between H_2_O_2_ and luminol. A microwell plate containing 16-phosphonohexadecanoic acid (16-PHA)-modified ZnO NPs was used as a platform for a series of immunoreactions with primary anti-CEA antibodies, secondary anti-CEA antibodies, and tertiary anti-CEA antibodies labeled with HRP. A 3-fold CL enhancement was possible with the aid of the ZnO NPs as opposed to a conventional ELISA method. The enhancement by the ZnO NPs was hypothesized to be due to their catalytic role in the radical generation and electron transfer processes of the CL reaction. The ZnO NP-aided ECL detection of CEA resulted in a linear range and LODs of 0.001–20 ng/mL and 0.001 ng/mL, respectively. In another ECL approach, vertically aligned ZnO NRs were used for the detection of choline [46]. ZnO NRs of ~80 nm in diameter were prepared on a glass substrate and crosslinked with 16-PHA. HRP and choline oxidase (COD) were then covalently attached to the ZnO NRs via EDC/NHS coupling to the carboxylic acid-terminated 16-PHA. The oxidation of choline by COD produced betaine aldehyde and H_2_O_2_. The CL intensity was found to be seven times higher when the COD/HRP enzymes were covalently linked rather than physically adsorbed to the ZnO NRs. This was supported by the lower Michaelis–Menten constant (K_m_) values of 0.062 mM versus 0.2 mM for the covalent versus noncovalent coupling cases of COD/HRP, respectively. The ECL intensity increased linearly with the concentration of choline in the range of 0.006–2 mM with a LOD of 0.0005 mM.

**Figure 8:**
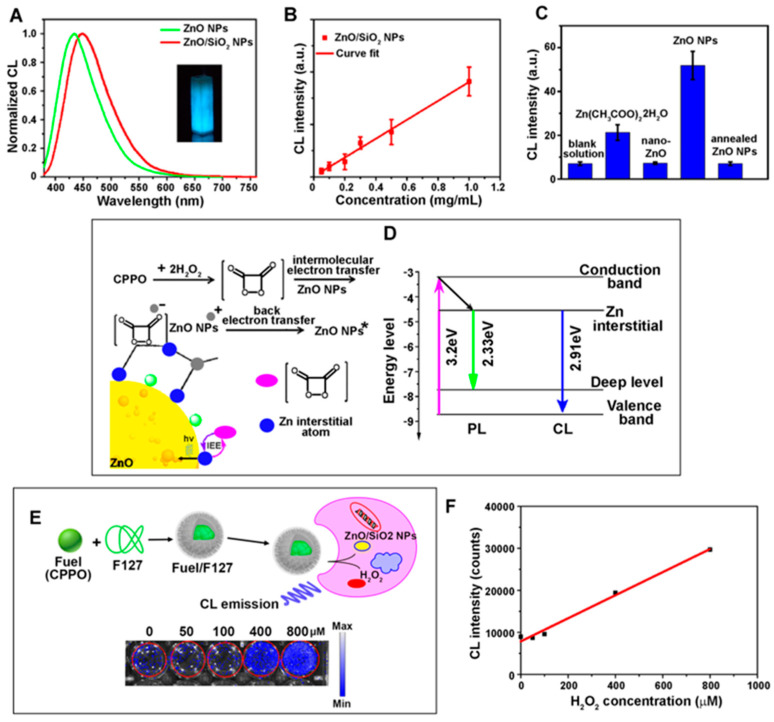
ZnO NPs coated with SiO_2_ were used as a CL probe in HeLa cell imaging. (**A**) The graph shows the CL spectra of the ZnO NPs and ZnO/SiO_2_ NPs in CPPO/H_2_O_2_ solution. The photograph of the CPPO/H_2_O_2_ solution taken in the dark after adding the ZnO/SiO_2_ NPs is shown in the inset. (**B**) The plot displays the CL intensity versus the concentration of the ZnO/SiO_2_ NPs. (**C**) The PL spectrum of the blank, Zn(Ac)_2_·2H_2_O, nano-ZnO NPs, ZnO NPs, and annealed ZnO NPs in the CL analysis system. The CL intensity induced in the CPPO/H_2_O_2_ solution is displayed in the bar graphs for the ZnO NPs as well as other controls such as the blank solution, Zn(CH_3_COO)_2_·2H_2_O, nano-ZnO, and annealed ZnO NPs. The ZnO NPs and Zn(CH_3_COO)_2_·2H_2_O exhibit CL enhancement, whereas no impact on CL is observed from the nano-ZnO and annealed ZnO NPs. From this, the interstitial Zn atoms were deduced to be engaged in the CL process. (**D**) The schematics illustrate the CL process of the ZnO NPs. CPPO reacts with H_2_O_2_, producing 1, 2-dioxetanedione. With the injection of the ZnO NPs, a chemically initiated electron exchange luminescence (CIEEL) occurs between the intermediate and surface interstitial Zn atoms of the NPs. The interstitial Zn defects are then excited and transited to the VB, emitting blue light. The ZnO NPs exhibit yellow PL emission due to DLE associated with the electron transition from the energy level of the interstitial Zn atom to that of Zn vacancy. The CL process affects the ZnO PL, causing a red-shift in PL. (**E**) The schematic illustration represents the use of the biocompatible ZnO/SiO_2_ NPs in CL-based cell imaging. The CL images were taken from HeLa cells cultured with the ZnO/SiO_2_ NPs for 6 h and then added to the CPPO mixture of different H_2_O_2_ concentrations, as specified in the bottom panel. (**F**) The CL intensities measured from the CL images in (**E**) are displayed. The CL intensity was directly proportional to the H_2_O_2_ concentration. Adapted with permission from Ref. [143], copyright (2020) Elsevier B.V.

ZnO NPs have also been employed as CL probes in quantitative cell imaging, as shown in Figure 8 [143]. ZnO NPs of ~5 nm in size were synthesized as a CL probe for imaging HeLa cells. The sol-gel-grown ZnO NPs exhibited PL, i.e., a yellow DLE at 535 nm. In the next step, bis(2,4,5-trichloro-6-carbopentoxyphenyl) oxalate (CPPO) and H_2_O_2_ were added to a solution of ZnO NPs. The CPPO/H_2_O_2_ reaction led to the formation of 1,2-dioxetanedione. A chemically initiated electron exchange luminescence (CIEEL) then occurred between 1,2-dioxetanedione and the interstitial Zn atoms on the ZnO NP surface. A blue CL emission centered at ~425 nm was subsequently generated from the excited, interstitial Zn defects. The CL processes were also examined by using SiO_2_-coated ZnO NPs (ZnO/SiO_2_ NPs). The SiO_2_ sheath was designed to passivate the ZnO NP surface and reduce its defect-related nonradiative recombination while also preventing the NP disintegration in downstream reactions. The CL with ZnO/SiO_2_ NPs resulted in a peak at 450 nm, red-shifted from the CL peak of the bare ZnO NPs. The DLE of ZnO/SiO_2_ NPs centering at 530 nm exhibited a longer lifetime than the DLE of the bare ZnO NPs. Regardless of the sheath’s presence, the CL intensity with ZnO NPs was found to be higher than that observed in the control samples, such as those with a chemiluminescent phosphor of Zn(CH_3_COO)_2_·2H_2_O. Quantum yields measured in einstein per mole (E/mol) for the systems of ZnO NPs and ZnO/SiO_2_ NPs were 6.2 × 10^−6^ and 3.72 × 10^−4^, respectively. The surface modification of the ZnO NPs with SiO_2_ resulted in a 60-fold increase in the observed CL. HeLa cells were successfully imaged using the CL reaction between ZnO/SiO_2_ NPs and CPPO/H_2_O_2_. The CL intensity was found to be directly proportional to the concentration of H_2_O_2_. It was also reported that ZnO/SiO_2_ NPs exhibited a low cytotoxicity. More than 90% of HeLa cells were viable, even when the concentration of the NPs used in a cell viability assay using 3-(4,5-dimethylthiazol-2-yl)-2,5-diphenyltetrazolium bromide (MTT) was as high as 500 µM.

### 2.5. Surface Evanescence, Whispering Gallery Mode, and Lossy-Mode Resonance

#### 2.5.1. Contributions of ZnO Nanomaterials in Various Guided- and Lossy-Mode Biodetection

ZnO nanomaterials of 0D, 1D, and 2D forms have all been utilized in optical waveguide-based biodetection. Due to their isotropic shape, 0D forms of ZnO nanomaterials are not suitable for directionally guiding optical signals. However, ZnO NPs can be fabricated on the surface of a conventional optical fiber to facilitate the surface evanescence wave-based detection of the optical fiber sensor. 1D ZnO nanomaterials, on the contrary, are effective both as optical waveguides and optical resonators. The subwavelength waveguiding nature of ZnO NRs and their applications in the enhanced fluorescence detection of various bioanalytes have already been discussed in the section on fluorescence. In addition, the highly crystalline, mirror-like, hexagonal facets of ZnO NRs can be an ideal cavity resonator for light waves [144,145,146,147,148,149,150]. Extensive research efforts on such properties of ZnO NRs have been made in optoelectronics and optics [90,111,119,151,152]. It is well known that resonant optical modes can exist inside the optical cavity of a ZnO NR in the form of Fabry–Perot modes (FPMs) and whispering gallery modes (WGMs). FPMs refer to the resonant modes from the electromagnetic (EM) waves confined between the two opposing facets either along the short or long direction of the NR. In contrast, WGM occurs from the resonance conditions fulfilled by completing a full loop around the six side facets along the short NR direction. Although FPM plays a dominant role in ZnO NRs, while WGM becomes the major mode for ZnO of a much larger size, WGM has been observed for ZnO NRs with diameters as small as 200 nm [149,150,151]. A thin layer of ZnO, as well as of ZnO/MoS_2_ composite, has been utilized for an entirely different type of EM resonance called lossy-mode resonance (LMR). LMRs, in this case, are produced under specific conditions by the ZnO or ZnO/MoS_2_ thin film that was coated on the etched surface of a conventional optical fiber [48,49]. Although some of these techniques are relatively newer to the field of optical biosensors than those methods covered elsewhere in this review, their applications have shown steadfast progress and have further expanded the usefulness of ZnO nanomaterials in optical biodetection. The following section will discuss such applications of ZnO nanomaterials that rely on the guided modes of a ZnO NP- or ZnO NR-coupled optical fiber sensor, ZnO NRs in a WGM sensor, and ZnO thin films in an LMR sensor.

#### 2.5.2. Applications of 0D/1D ZnO Nanomaterials in Surface Evanescent Wave Biosensors

As introduced earlier, optical waveguides such as optical fibers can confine the EM fields of light waves and guide them through total internal reflection (TIR). As light propagates through an optical waveguide with its characteristic dimension at the nanometer range, a significant portion of the EM is carried in the form of surface evanescent waves. The intensity of the surface evanescent wave decays exponentially with the distance from the waveguide surface into the surrounding medium. Even a small change in the medium in the vicinity of the waveguide can significantly affect the evanescent wave. Most optical fibers used in biodetection have their surfaces functionalized with bioreceptors for the subsequent binding of target analytes in solution. The binding of analytes causes a change in the refractive index and alters the propagation characteristics of the surface evanescent wave, serving as a source of the analyte signal. The high refractive index of ZnO, along with the well-established straightforward synthesis methods to create ZnO nanomaterials, makes them suitable for this detection scheme [1,89,90,91].

ZnO NPs linked to a conventional optical fiber of plastic-clad silica were demonstrated for urea detection by monitoring the absorbance of the surface evanescent wave [47]. A polyaniline–ZnO (PANI-ZnO) nanocomposite was prepared from ZnO NPs of ~26 nm that were synthesized by a co-precipitation method. Urease (Urs) was then immobilized to the PANI-ZnO matrix and attached to the unclad portion of the optical fiber. Under urea, ammonia and carbamate were formed from the urea reaction catalyzed by Urs. The evolution of ammonia altered the refractive index of the ZnO NP-modified optical fiber, and the rate of ammonia evolution measured by absorption at ~250 nm changed with the concentration of urea. The linear range of the ZnO NP-modified optical fiber sensor was reported to be 10 nM–1 M of urea with a LOD of 10 nM. Similarly, a ZnO NR-modified optical fiber was used to detect uric acid [15,153]. For this, ZnO NRs were coated onto the tapered surface of a plastic optical fiber (POF) via the sol-gel immersion method [15]. The tapered geometry was responsible for allowing a higher portion of the evanescent waves to travel into the cladding. The electrostatic interaction between the ZnO NRs and uric acid induced a change in transmitted light through the optical fiber. The transmitted light was measured with and without the presence of uric acid, whose signals were converted into an output voltage by a Si photodetector. With a higher concentration of uric acid, the refractive index of the solution also increased. Consequently, the output voltage of the uric acid sensor yielded a linear range and a LOD of 0–500 ppm and 5.6 ppm, respectively.

#### 2.5.3. Applications of 1D ZnO Nanomaterials in Whispering Gallery Mode Biosensors

Highly crystalline hexagonal facets of ZnO NRs, typically attained by gas-phase synthesis methods such as CVD and metal organic chemical vapor deposition (MOCVD), are conducive to those needed in an ideal optical resonator [1,109,118,154,155,156]. ZnO NRs can act as WGM resonators since light in a ZnO NR can be effectively confined and circulated within the NR cavity via multiple TIRs around its short axis. During this process, light waves at a resonant frequency constructively interfere without experiencing significant losses, whereas those of other frequencies annihilate due to deconstructive interferences. For use as a WGM resonator biosensor, ZnO NRs are first modified with a bioreceptor with a specific affinity to the bioanalyte of interest. The presence of the bioanalyte in the vicinity of the ZnO NR WGM resonator changes the effective refractive index of the medium and, subsequently, the resonant spectrum of the sensor. These changes in the resonant spectrum, such as mode shifting, mode splitting, and mode broadening, are monitored to correlate with the bioanalyte concentrations in a label-free and real-time manner [44]. Mode shift in the resonant wavelength is the most common mechanism for ZnO NR WGM resonators and has broad applicability to a range of analytes. The self-referencing mechanisms of mode splitting and mode broadening can be useful when it is important to isolate the environmental effects, such as the change in the temperature, pressure, and humidity, from the analyte signals.

**Figure 9:**
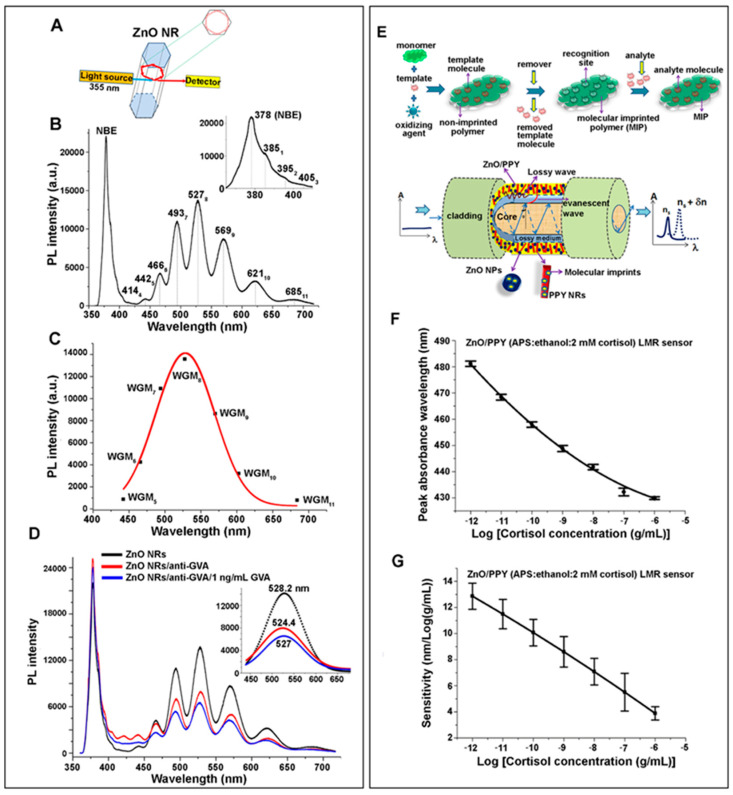
Examples of WGM and LMR biosensors fabricated from ZnO nanomaterials are shown. (**A**) The schematic illustrates a ZnO NR-based WGM biosensor and the WGM optical paths generated inside the NRs. (**B**) The plot corresponds to the PL spectrum of the Mn-doped ZnO NRs vertically oriented on a Si substrate. The PL spectrum shows the NBE emission at 378 nm, as well as multiple broad DLE peaks between 450–650 nm. WGMs of the DLEs were formed inside the optical resonators of the ZnO NRs, as annotated for each WGM peak. (**C**) The Gaussian fitting result of the WGM data in (**B**) is displayed in the plot. The WGM emission maximum occurred at 528.2 nm for the ZnO NRs, whose peak was blue-shifted to 524.4 nm upon anti-GVA immobilization to the NRs and subsequently red-shifted to 527 nm after further incubation with GVA. (**D**) The PL spectra of the ZnO NRs, as well as those treated with anti-GVA and subsequently with 1 ng/mL GVA, are presented. The inset belongs to the Gaussian-fitted WGM peaks. (**A**–**D**) Adapted with permission from Ref. [44], copyright (2020) Elsevier B.V. (**E**) The pictorial representation shows a lossy-mode resonance (LMR) biosensor fabricated from a ZnO thin film with polypyrrole (PPY) prepared with a molecular imprinting polymer technique. The LMR sensor was subsequently used in cortisol detection. (**F**) The plot displays the absorbance wavelength of the LMR peak as a function of cortisol concentration. A blue shift of 51.23 nm in the LMR absorbance wavelength was observed for the cortisol concentration range of 10^−12^–10^−6^ g/mL. (**G**) The sensitivity for the different cortisol concentrations is shown for the LMR sensor prepared with 20% ZnO/PPY. The sensitivity was evaluated from the sensor calibration curve by taking the derivative of the curve fit equation with respect to cortisol concentration. A maximum sensitivity of 12.86 nm/Log (g/mL) was yielded for 10^−12^ g/mL of cortisol. This rate of change in the absorbance wavelength decreased with increasing cortisol concentration. (**E**–**G**) Adapted with permission from Ref. [48], copyright (2016) Elsevier B.V.

A WGM resonator of vertically grown, Mn-doped ZnO NRs on a Si substrate was successfully demonstrated as an immunosensor for GVA proteins [44]. Upon irradiation with 355 nm light at an incidence angle of ~20° with respect to the underlying Si substrate, the Mn-doped ZnO NRs with an NR diameter of ~200 nm produced an NBE emission at 378 nm and broad DLEs at 450–650 nm. These emissions of the NRs further formed quasi-WGMs as they were reflected from each facet of the hexagonal NR and spirally propagated under a resonant condition. These quasi-WGMs were characterized in the UV and visible regions, from which the most intense emission was found at ~528 nm. Figure 9A–C display the formation of WGMs inside the ZnO NRs, as well as the different WGM peaks observed. The immobilization of the anti-GVA antibodies (anti-GVA) resulted in a blue shift of this WGM peak, and the subsequent binding of GVA to anti-GVA led to an opposing red shift of the WGM peak. The ZnO NR WGM resonator was sensitive to GVA proteins in the concentration range of 1–200 ng/mL. A representative GVA detection result of the ZnO NR WGM biosensor is shown in Figure 9D for the GVA concentration of 1 ng/mL.

#### 2.5.4. Applications of 2D ZnO Nanomaterials in Lossy-Mode Resonance Biosensors

An optical waveguide coated with materials can produce an attenuation band in its transmission/absorption spectra under certain conditions due to the resonant coupling between guided and lossy modes called LMR [157,158,159,160]. These conditions are influenced by factors such as the incident light wavelength and coating thickness. Therefore, the propagation of light in the optical waveguide at certain wavelengths and incident angles can have attenuation maxima for specific thickness values of the cladding that change before and after analyte binding. This property of LMR enables the label-free and real-time monitoring of bioanalytes. LMR-based detection offers several advantages over other optical resonance techniques, such as SPR, which will be detailed in the next section of SPR. The requirement for LMR excitation is considered to be less demanding relative to SPR. LMR can be excited by both transverse magnetic (TM) and transverse electric (TE) polarized light. LMR can also generate multiple resonances in the same transmission spectrum [159]. In addition, LMR can be produced by many materials as long as the real part of the material’s permittivity is positive and the absorption coefficient is low [160]. Hence, the material choice for LMR biosensor platforms can be more inclusive, where dielectrics, polymers, and TCOs, including ZnO, can all be used. This can make the applications of LMR-based approaches convenient and flexible in biosensing.

ZnO thin films integrated on a multimode plastic-clad silica fiber were shown for the LMR-based detection of p-cresol and cortisol [48,49]. An LMR sensor was developed using a ZnO/polypyrrole (PPY) nanocomposite as the probe to detect cortisol, as displayed in Figure 9E–G [48]. ZnO and PPY were chosen since their conductivity can improve the overall LMR sensing properties. For LMR detection, a 12 nm thick ZnO film was thermally evaporated on the unclad core of a silica fiber. The ZnO-coated optical fiber was then coated with PPY, which served as a molecular imprinted polymer (MIP) for cortisol binding. The presence of cortisol led to changes in absorbance in terms of an increased peak intensity and a blue-shifted peak wavelength. When the shift in the peak wavelength was used for cortisol detection, the ZnO/PPY thin-film LMR sensor provided a linear range of 10^−11^–10^−7^ g/mL and a LOD of 25.9 fg/mL. In another LMR-based detection, a ZnO/MoS_2_-cladded optical fiber was coated with a 1.2 µm thick MIP of PMMA and subsequently used for the detection of p-cresol [49]. In this case, PMMA served as an MIP since MMA in PMMA could form hydrogen bonds with the OH functional group in p-cresol. The binding of p-cresol led to changes in the refractive index and LMR conditions. The resonance wavelength monitored between the range of 300–700 nm showed a dip in intensity and a peak shift in the absorption spectrum. With increasing p-cresol concentration, a red shift in LMR wavelength was observed. This was correlated to a sensor operation range of 0.028–1000 µM and a LOD of 28 nM for p-cresol.

### 2.6. Surface Plasmon Resonance

#### 2.6.1. Overview of Surface Plasmon Resonance in Biodetection

Surface plasmons (SPs) are collective excitations of conduction electrons at the interface between a metal and a dielectric that appear in two forms depending on their conditions: localized surface plasmon polaritons (LSPs) and propagating surface plasmon polaritons (SPPs). Since the size of a nanomaterial is significantly smaller than the wavelength of the incident light, the electron oscillations are restricted to the surface of the nanostructure. This confinement produces a highly localized and amplified EM field at a particular resonance wavelength, whose phenomenon is known as localized surface plasmon resonance (LSPR). The phenomenon of LSPR has been exploited to increase the detection signals in absorption- and fluorescence-based assays, which were discussed previously. LSPR also serves as the underlying mechanism of the surface enhancement reported in Raman scattering. These applications, in which metal NPs of a few to several nm in diameter are used without a supporting structure of a 2D waveguide or prism, will be discussed later in the section on surface-enhanced Raman scattering.

In contrast to LSPs, SPPs refer to surface electron oscillations that propagate along a thin metal layer fabricated on an optical waveguide or prism. Transverse magnetic (TM)-polarized light incident upon such a layered structure produces evanescent waves that decay exponentially by attenuated total reflection (ATR) [161]. SPPs are then formed in the metal film and further generate propagating electron density waves called surface plasmon waves (SPWs) along the metal–dielectric interface. These SPWs carry a strong EM field by wavevector-matching between the incoming light and SPWs. This phenomenon is referred to as surface plasmon resonance (SPR) [162,163,164,165]. Hence, in this SPR section of the review, research efforts based on the generation of SPWs as a transduction mode in bioanalyte sensing are discussed.

**Figure 10:**
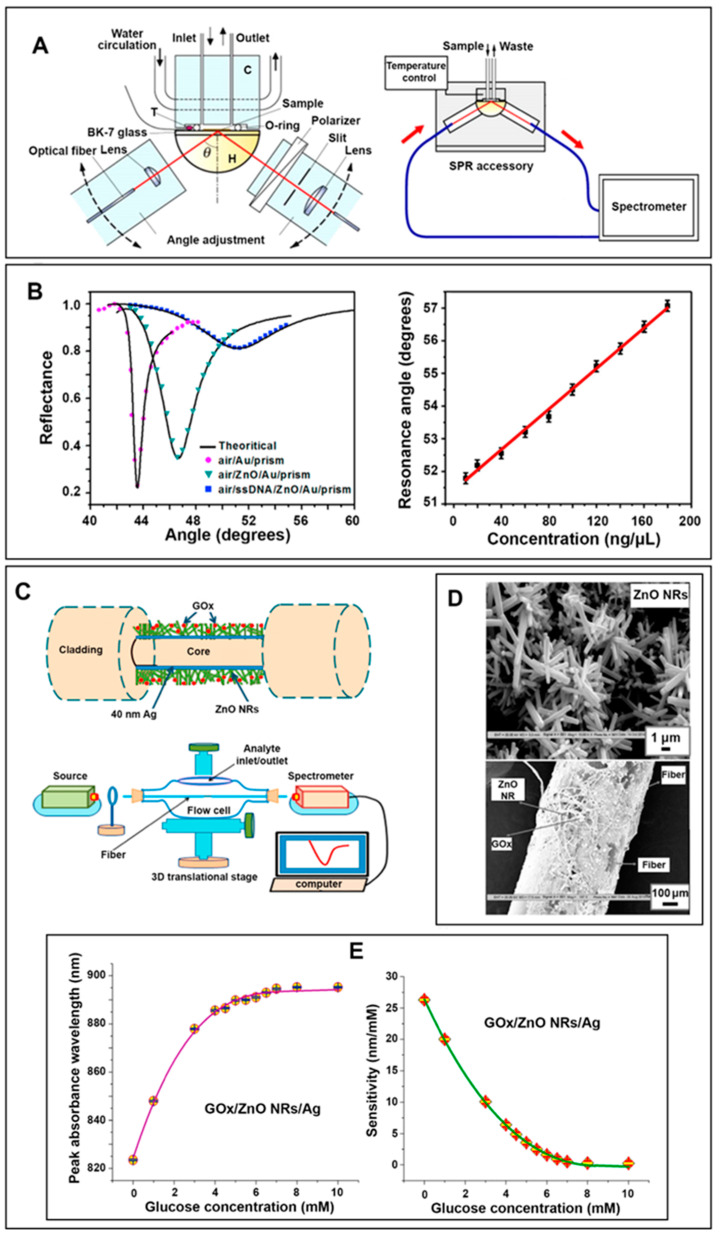
Exemplar uses of ZnO nanomaterials in both prism- and optical fiber-based SPR biosensors are presented. (**A**) The schematic diagram depicts a typical SPR detection setup involving a Kretschmann-type attenuated total reflection (ATR) attachment. C, H, and T indicate the flow cell, base thermistor, and hemicylinder prism, respectively. Adapted with permission from Ref. [173], copyright (2020) American Chemical Society. (**B**) The left plot is the SPR reflectance curves obtained from the platforms of air/Au/prism, air/ZnO/Au/prism, and air/ssDNA/ZnO/Au/prism used for the detection of *N. meningitidis* DNA. The plot on the right is the calibration curve created from the SPR curves of the air/ssDNA/ZnO/Au/prism platform upon ssDNA hybridization with different concentrations of complementary DNA. The interaction between the probe ssDNA with increasing concentrations of target DNA resulted in a continuous shift in SPR angle towards a higher angle. The SPR biosensor exhibited a linear response range of 10–180 ng/μL *N. meningitidis* DNA and a LOD of 5 ng/μL. Adapted with permission from Ref. [50], copyright (2015) Elsevier B.V. (**C**) The schematic diagram illustrates an optical fiber SPR setup used for glucose detection. An unclad portion of plastic-clad silica fiber was coated with a Ag layer onto which ZnO NRs were synthesized. The sensing probe was then immobilized with GOx. (**D**) The SEM images display the hydrothermally grown ZnO NRs in the top panel and the silica fiber surface modified with Ag/ZnO NRs/GOx in the bottom panel. (**E**) The left plot corresponds to the changes in peak absorbance wavelength as a function of glucose concentration. The shift in the peak absorbance wavelength was ~72 nm in the glucose concentration range of 0–10 mM. The plot on the right shows the sensitivity of the sensor for different glucose concentrations. The sensitivity was calculated from the change in the peak absorbance wavelength per unit change in glucose concentration. (**C**–**E**) Adapted with permission from Ref. [14], copyright (2016) Elsevier B.V.

SPR excitation requires a coupling medium to provide the required photon momentum along the interface, which is typically attained by a thin film deposited on a high-index prism, a grating, or a waveguide such as the well-known Kretschmann–Reather (KR) configuration. An example of a typical SPR measurement setup involving a prism configuration is shown in Figure 10A. Both the angle and the wavelength of incident light in SPR measurements should satisfy specific conditions, leading to the excitation of surface plasmons. In fact, only TM-polarized light can be used to induce SPR excitation. In addition, the real part of the dielectric constant for the thin film must be negative, and its value should be higher than both the imaginary part of the thin film and the real part of the surrounding medium. Hence, only conductive metal or metal oxides can excite SPR. Once these conditions are met, the incident light is absorbed by the thin metal film via SPR, and its transmission spectrum shows a resonance dip. The performance of SPR biosensors is affected by the properties of the thin film deposited on the optical waveguide or prism [166,167]. As such, SPR biosensors detect molecular binding events by monitoring the change in the refractive index of the dielectric layer near the metal surface. These changes can be due to the binding of bioanalytes directly to the metal surface or to bioreceptors immobilized at the metal–dielectric interface. The binding of a bioanalyte and its concentration are monitored through the phase shift [168], resonant angle [169], and intensity [170], as well as the wavelength of the reflected beam [171]. As SPR spectroscopy can monitor real-time changes of bioanalytes with high sensitivity in a label-free manner, extensive research efforts have been made to develop various nanomaterial-based SPR biosensors [162,163,164,165].

#### 2.6.2. Contributions of ZnO Nanomaterials in Surface Plasmon Resonance-Based Biodetection

ZnO is transparent in the visible range due to its large bandgap, and, unlike the metals of Au or Ag, it does not have CB electrons available for SPR at visible wavelengths [1]. Despite this, ZnO is widely employed in conjunction with noble metals to enhance the detection capability of SPR sensors, both in prism and optical fiber configurations [1,172]. The presence of ZnO in SPR sensors facilitates biodetection in multiple ways. The light trapping and collecting abilities of ZnO are beneficial for the generation of more SPs from noble metals at the sensor surface. ZnO nanomaterials can also act as a high-index medium at the SPR sensor interface that enhances the SPR signal from bioanalytes whose binding alone may not elicit large enough changes in the refractive index for detectable signals. Having a high isoelectric point of ~9.5, ZnO nanomaterials promote the attachment of most biomolecules to the sensor surface via strong electrostatic interactions [1]. This biochemical property of ZnO, along with the high surface area inherent to the nanomaterial, enables the recruitment of more biomolecules on the sensor surface for better reactions with bioanalytes and, subsequently, the detection of larger SPR signals. ZnO nanomaterials also contribute to the chemical stability of the metals in SPR sensors. For example, the incorporation of ZnO nanomaterials in Ag-based SPR sensors can protect the metal from its low chemical stability [172].

#### 2.6.3. Applications of 0D/2D ZnO Nanomaterials in Surface Plasmon Resonance Biosensors

ZnO NPs linked to Au nanomaterials were utilized for the detection of human IgM, human IgG (hIgG), and rabbit IgG [25,174,175]. For example, ZnO NPs coated with Au (ZnO NPs/Au) were developed as an SPR sensing surface to detect hIgG [25]. The ZnO NPs were first immobilized on a Au film-covered prism and then coated with polydopamine (PDA) to increase the hydrophilicity and biocompatibility of the sensor surface. A sandwich immunoassay was carried out on the SPR biosensor using anti-IgG (Ab_1_) as a capture antibody and Au NR–streptavidin–biotin–anti-IgG (GSAB-Ab_2_) as a secondary amplification unit of the SPR signal. The excitation of SPRs was obtained by a TM-polarized white light source, and changes in the SPR wavelength at 520 nm were monitored upon the addition of hIgG. The binding of hIgG to the antibody complexes resulted in a shift of the SPR wavelength. A maximum shift of 13.54 nm was attained from 40 µg/mL hIgG. The hIgG detection range and LOD of the ZnO NP/Au SPR biosensor were determined to be 0.0375–40 µg/mL and 0.0375 µg/mL, respectively. In a different research effort, 2D ZnO thin films were demonstrated for the SPR-based detection of *Neisseria meningitidis* (*N. meningitidis*) and streptavidin [50,173]. In the example of *N. meningitidis* detection displayed in Figure 10B, a ZnO thin film containing a capture ssDNA was first configured on a Au-coated prism [50]. Subsequent hybridization reactions between the analyte DNA of *N. meningitidis* and its complementary ssDNA probe at the ZnO thin film/Au sensor surface led to refractive index-derived changes in terms of both the intensity and resonant angle of the reflected light. With increasing *N. meningitidis* DNA concentration, a continuous shift towards higher angles was observed in the SPR reflectance curve. The ZnO thin film/Au SPR sensor provided a linear range of 10–180 ng/µL and a LOD of 5 ng/µL for the *N. meningitidis* detection.

#### 2.6.4. Applications of 1D ZnO Nanomaterials in Surface Plasmon Resonance Biosensors

In addition to the prism and waveguide approach, strategies to integrate SPR with optical fiber technology were made to capitalize on the signal transmission and guidance capability of an optical fiber in SPR detection. Optical fiber-based SPR sensors offer many advantages. Relative to the conventional planar SPR setup discussed before, an optical fiber device can be easily miniaturized, and no loss in the optical signals occurs through an underlying substrate [163,176,177,178]. Despite these advantages of optical fiber SPR detection, the technique currently faces difficulties in detecting bioanalytes of low molecular weights and low concentrations. The technique also suffers from lower sensitivity due to the lower mass that can bind to the fiber relative to the conventional SPR platform [179]. To this end, ZnO NRs were incorporated into optical fiber SPR sensors to improve the detection capability.

One-dimensional ZnO NRs coupled with plasmonic nanomaterials were demonstrated as optical fiber SPR sensors for the detection of glucose and PSA [14,176]. For the example of glucose detection, ZnO NRs were prepared hydrothermally into diameters of 120–250 nm on the surface of a Ag film-modified silica fiber [14]. The ZnO NR-coupled optical fiber surface was then treated with GOx. Figure 10C–E present the ZnO NR-coupled optical fiber SPR detection scheme, as well as the results of glucose detection. In the presence of glucose, the formation of the reaction products, such as gluconolactone and H_2_O_2_, caused an increase in the effective refractive index at the optical fiber interface, along with a red shift in the SPR wavelength measured at ~823 nm. The presence of ZnO NRs promoted an effective electrostatic recruitment of GOx to the sensor and enhanced the surface activity of the enzyme. The presence of the high-index material of ZnO NRs around the Ag-coated unclad fiber facilitated the sensitivity of the sensor probe. Owing to these sensor improvements, a maximum peak shift of 72 nm was obtained from 10 mM glucose. The dynamic range and LOD of the optical-fiber SPR sensor were reported to be 0–10 mM and 0.012 mM, respectively, for glucose.

### 2.7. Surface-Enhanced Raman Scattering

#### 2.7.1. Overview of Surface-Enhanced Raman Scattering in Biodetection

Raman scattering offers a powerful analytical tool for analyte detection, capable of providing the chemical fingerprints of an analyte molecule [180]. For most bioanalytes, such as proteins, nucleic acids, and lipids, the spectral fingerprint regions lie in the Raman shift window between 800 and 1800 cm^−1^ [181,182]. Raman signals, however, are inherently more difficult to detect than other optical signals, such as fluorescence, due to their very weak scattering cross-section of 10^−28^–10^−30^ cm^2^ per molecule [183]. Surface-enhanced Raman scattering (SERS) was developed to mitigate this issue. SERS refers to the enhanced Raman scattering from analytes adsorbed on plasmonic nanostructures such as Au, Ag, Pd, Pt, and Cu [184,185,186,187,188,189,190]. Two mechanisms, EM enhancement and chemical enhancement (CE), are usually attributed to SERS [191,192]. EM enhancement due to LSPR is dominant for noble metal SERS systems, whereas CE is dominant for semiconductors, metal oxides, and carbon-based materials since it involves charge transfer (CT) processes between the molecular orbitals of an analyte and the Fermi level of an SERS-active material.

When incident light interacts with a plasmonic NP, LSPR with a highly enhanced EM field is triggered on the spatially localized region around the NP. This enhances the Raman scattering signals of nearby analytes through EM enhancement, which is proportional to the fourth power of the field due to the enhancement of the excitation field at the metal NP surface, as well as that of the Raman-scattered light from the analyte adsorbed on the NP [185,186,188,190,193]. Typically, the enhanced field around the metal NP decays at a relatively short length of 5–30 nm [194,195]. It was found that a significantly enhanced electric field also exists in the gap between metal NPs called SERS hotspots [195,196]. SERS hotspots also exist at the sharp edges and corners of small topographic features of less than 10 nm in size. A higher SERS enhancement is expected from a nanomaterial that presents more of these sharp edges and corners. For example, for the Au nanosystems of different shapes, the order in SERS EF from highest to lowest was reported as Au nanostars, Au NRs, and Au NPs [197]. Later, SERS was additionally observed with high reproducibility on non-metallic nanostructures, such as ZnO and a related TCO of TiO_2_ [198,199,200,201]. For the SERS applications of these semiconducting nanomaterials, CT processes responsible for the CE mechanism were recognized to be also important [192]. CT can occur in various pathways [202,203]. A common route is the electron transfer from the highest occupied molecular orbital (HOMO) of an analyte molecule to the unoccupied state above the Fermi level of a nanomaterial or that from the nanomaterial’s occupied state below its Fermi level to the lowest unoccupied molecular orbital (LUMO) of the molecule. Other routes that can contribute to CT involve the case of exciton resonance from the generation of an electron–hole pair in the nanomaterial, as well as the case of resonance in newly created energy levels from a nanomaterial–analyte complex. These CT processes alter the polarizability and electron density of the analyte, leading to increased Raman scattering via the CE mechanism [185,186,188,190,193].

The enhancement factor (EF) is one of the key parameters in quantifying enhanced Raman signals and assessing the effectiveness of an SERS platform. The general formula for EF can be written as (I_SERS_/N_Surf_)/(I_RS_/N_Vol_) [204]. I_SERS_ and I_RS_ are the Raman intensities measured under SERS and normal conditions, respectively. N_Surf_ is the average number of adsorbed molecules on an SERS platform, and N_Vol_ is the average number of molecules in the regular Raman scattering volume. SERS substrates consisting of the commonly used Ag NPs were reported to induce EFs as high as 10^14^–10^15^ for the model analytes of R6G and crystal violet [205,206]. The EF in SERS is proportional to the distance (d) between the NP and the analyte molecule as ~d^−12^ [180]. The characteristic LSPR wavelength of the NP, as well as the EF from the NP, are strongly governed by the size, shape, and chemical composition of the NPs [181,182]. Hence, it is possible to optimize the EF in SERS bioapplications by controlling the NP parameters during its synthesis in such a way that a characteristic LSPR wavelength of the NP is matched well with the excitation wavelength used for Raman scattering. SERS measurements can be carried out both with and without the use of an SERS label. A large variety of SERS tags are available that produce high-intensity spectral peaks, and their structure typically consists of an NP core functionalized with a Raman reporter molecule [207,208].

#### 2.7.2. Contributions of ZnO Nanomaterials in Raman Scattering-Based Biodetection

ZnO nanomaterials have been successfully incorporated into SERS platforms for enhancing the SERS signals even further [128,187,209,210]. They have been used both as standalone as well as hybrid SERS platforms, the latter being more frequently found in the literature. As for ZnO nanomaterials used without noble metals, it was shown that the C-S stretching mode of 4-mercaptopyridine (4-Mpy) on the surface of 20 nm ZnO NPs exhibited a large peak shift to 727 cm^−1^, as well as an increased intensity for the peak at 1119 cm^−1^, relative to those measured from 4-Mpy in solution [198]. The presence of the ZnO NPs enabled an EF of 1 × 10^3^, which was hypothesized to stem from the CE mechanism owing to the increased polarizability of 4-Mpy upon chemisorption and photon-driven CT. In another work, calcined ZnO NPs of ~400 nm in size were used in Pb^2+^ detection, whose concentrations were measured by a Raman tag of methylene blue (MB). MB was incorporated into the DNA hybridization scheme for which, under Pb^2+^ ions, a trigger DNA piece formed a duplex with its complementary ssDNA that was linked to the calcined ZnO NPs [211]. The employment of the calcined ZnO NPs enhanced the Raman signal of MB at 1625 cm^−1^ relative to the case of uncalcined ZnO NPs. This enhancement was hypothesized to be due to the narrower band gap (3.05 eV) of the calcined ZnO NPs relative to the uncalcined NPs (3.21 eV). The narrower band gap promoted CT efficiency and SERS enhancement by having its CB closer in energy to the HOMO of MB. Although the analytes targeted in these initial studies were chemical species rather than biomolecules, these research efforts underscore the potential use of ZnO nanomaterials as a standalone Raman-enhancing element to facilitate the detection of analyte signals via CE. However, the SERS performance of a stand-alone semiconductor is usually limited to the EF of 10^2^–10^5^, and hence, different strategies of bandgap- and defect-engineering were employed to improve their SERS capability via CE [182,198,201,212].

A hybrid SERS platform can offer combined benefits from a noble metal and ZnO. In ZnO–metal hybrid applications, strong light absorption and Raman excitation occur in the visible to near-infrared (NIR) region owing to the EM enhancement through the metal. In addition, CT can occur between the Fermi level of ZnO and that of a metal [210,213,214]. The formation of a Schottky barrier at the ZnO–metal interface results in a strong electric field due to the accumulation of electrons at the interface [214]. Hence, the combined enhancement through both the EM and CE mechanisms can be exploited to significantly improve SERS sensors. Due to these reasons, ZnO–metal hybrid structures were developed for a number of SERS-based biodetection applications [27,52,53,54,55,213,214,215,216,217,218].

#### 2.7.3. Applications of 0D ZnO Nanomaterials in Surface-Enhanced Raman Sensors

ZnO NPs of ~10 nm in size were prepared for use in the Raman-based detection of subcellular biomolecular cancer signals [51]. During the NP synthesis via laser ablation, the atmospheric conditions of the reactor were controlled to generate different surface defects, such as oxygen vacancies, zinc vacancies, zinc interstitials, and adsorbed oxygen. The varying compositions of these defects determined the net charge of the ZnO NPs. The resulting ZnO NPs were first used to detect two molecules of positively charged 4-aminothiophenol (4-ATP) and negatively charged 4-mercaptobenzoic acid (4-MBA) by forming oppositely charged ZnO NP–analyte molecule pairs. The EFs obtained for 4-ATP and 4-MBA were 1.4 × 10^6^ and 1.97 × 10^6^, respectively, when they were paired with the oppositely charged relative to the neutral ZnO NPs. The oppositely charged ZnO NP–analyte molecule pairs resulted in greater binding of the analyte to the NPs, which, in turn, promoted SERS signal enhancement via CT. The use of the ZnO NPs was then extended to cells such as those of pancreatic cancer (AsPC1), breast cancer (MDAMB231), and non-cancer fibroblasts (NIH3T3). The cells were incubated with the ZnO NPs for cell uptake through endocytosis and diffusion. The cells were at least 95% viable after a 24 h incubation with ZnO NPs. Different subcellular components, such as negatively charged DNA, negatively charged lipids, and positively charged oncoproteins, were targeted by ZnO NPs. The Raman signals were then monitored at 1334, 1454, and 834 cm^−1^ for DNA, lipids, and proteins, respectively. Compared to the neutral NPs, the use of positively charged ZnO NPs for DNA binding resulted in a SERS enhancement of ~7-, 5-, and 3-fold for the cells of AsPC1, MDAMB231, and NIH3T3, respectively. Similarly, by using the positively charged ZnO NPs, the SERS signals of the lipids in the AsPC1, MDAMB231, and NIH3T3 cells were enhanced by ~24-, 22-, and 4-fold, respectively. For the detection of the oncoproteins, the use of negatively charged ZnO NPs resulted in a signal enhancement of approximately ~7-, 16-, and 3-fold from the AsPC1, MDAMB231, and NIH3T3 cells, respectively.

SERS detection was realized, even for a system that did not include plasmonic metal NPs [27]. In this case, core/shell QDs of CdSe/ZnO were utilized to enhance the Raman signals of BSA. The strong SERS-like behavior of the CdSe/ZnO QDs was caused by the changed local EM field through FRET. The energy transfer was explained to occur from the CdSe-based donor-excited states to a proximal ZnO acceptor through nonradiative dipole–dipole coupling. It was determined that four monolayers of ZnO around the CdSe core resulted in an effective surface passivation of the core and the strong signal enhancement of BSA. Eight characteristic peaks of BSA were detected with the aid of the CdSe/ZnO QDs, as opposed to only four weak peaks from BSA prepared on a Si substrate. The Raman platform of CdSe/ZnO QDs in BSA detection led to a LOD of 2.5 × 10^−6^ M.

#### 2.7.4. Applications of 1D ZnO Nanomaterials in Surface-Enhanced Raman Sensors

**Figure 11:**
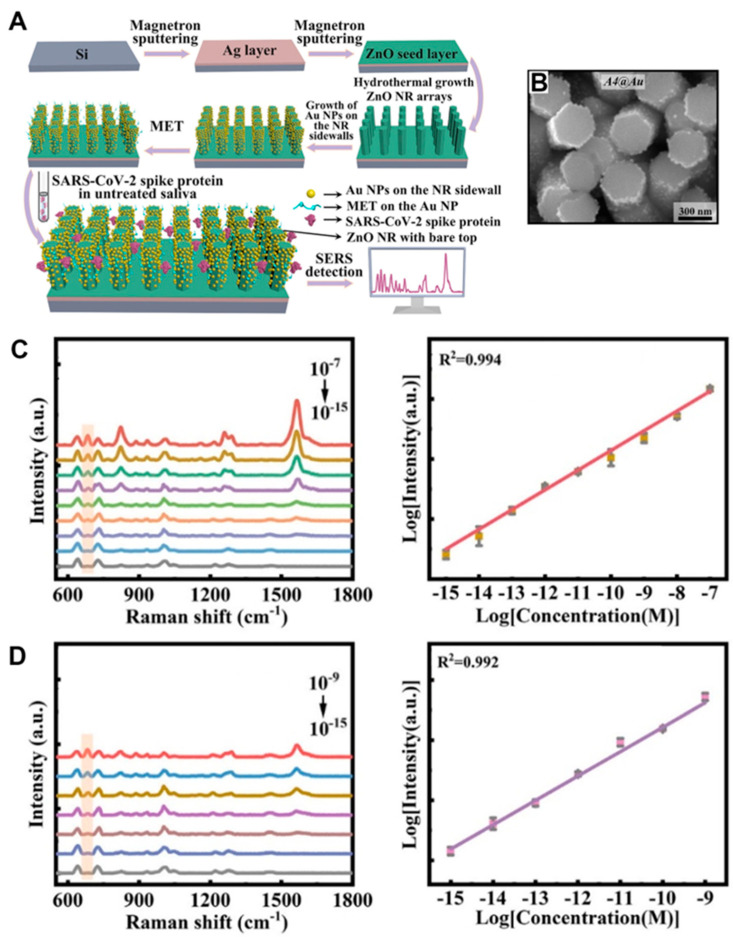
(**A**) The schematic diagram depicts the fabrication process for an SERS biosensor to detect the SARS-CoV-2 spike protein. The SERS sensor platform was constructed by synthesizing vertical ZnO NRs on a Ag layer and decorating the NR side walls with Au NPs. The Au NPs on the sensor platform were then modified with MET to promote the capture of the spike protein. (**B**) The top-view SEM image displays Au NPs decorating the side walls of the ZnO NRs. Five different SERS substrates of Au NP/ZnO NRs (A1 to A5) were used in the study by varying the average diameters of the ZnO NRs: ∼155, 205, 240, 349, and 435 nm. The substrate shown in the SEM panel corresponds to A4 with an NR diameter of ~349 nm, which was reported to yield the best SERS signals. (**C**) The plots display the Raman spectrum of the SARS-CoV-2 spike protein in PBS using the SERS substrate in (**B**). The Raman signals measured at 680 cm^−1^ were then used to create the Log–Log plot of Raman intensity versus protein concentration. Each error bar indicates the standard deviation of five different measurements from a single SERS substrate. (**D**) The detection of the SARS-CoV-2 spike protein in untreated saliva was carried out on the SERS substrate. The Raman intensity and Log–Log plots are provided for the saliva samples. Adapted with permission from Ref. [52], copyright (2023) Elsevier B.V.

ZnO NRs can be straightforwardly grown on different substrates that can be readily processed as SERS platforms [209,219]. This capability has been used in numerous SERS efforts to detect bioanalytes such as λ-DNA, lactate, dopamine, oxycodone, hemoglobin (Hb), pioglitazone (PIO), phenformin (PHE), and severe acute respiratory syndrome-related coronavirus-2 (SARS-CoV-2) spike protein [6,52,53,213,214,217,218]. An SERS platform of Au NP-ZnO NRs on a Ag film-coated Si wafer was developed in a study to detect SARS-CoV-2 spike protein [52]. During SERS measurements, the evanescent field on the surface of ZnO NRs was enhanced by the highly reflective Ag film, as well as the bare top facet of the vertically grown ZnO NRs. The enhanced evanescent field of the ZnO NRs promoted the LSPR excitation of the Au NPs, intensifying the EM field for SERS. At the same time, the highly reflective Ag film prevented the underlying Si substrate from absorbing the incident light, as well as the analyte signal. 2-mercaptoethanol (MET) coupled to Au NPs was used to capture SARS-CoV-2 spike protein while reducing other interfering signals present in saliva. Using this strategy, the LODs determined for the SARS-CoV-2 spike protein were 3.6 × 10^−17^ M in phosphate-buffered saline (PBS) and 1.6 × 10^−16^ M in untreated saliva. Figure 11 displays the SERS sensor fabrication process, the morphology of the ZnO NRs with Au NPs used as an SERS substrate, and the Raman detection results for the SARS-CoV-2 spike protein in PBS, as well as saliva samples. In a different study, Au-coated ZnO NRs were used as an SERS substrate for MB detection [204]. A ZnO seed layer was first prepared on a Si wafer via atomic layer deposition (ALD), after which vertically aligned ZnO NRs were hydrothermally grown to a length of 300–400 nm and a diameter of 30–40 nm. The ZnO NRs were then sputter-coated with Au. It was hypothesized that ~5 nm gaps between the adjacent Au islands of the Au-coated ZnO NRs can serve as SERS hotspots, leading to SERS enhancement. Thicker Au layers, after prolonged sputtering beyond 30 s, diminished these gaps, resulting in a decreased SERS signal. Upon optimization, Au-coated ZnO NRs were employed for the detection of MB by monitoring the Raman shifts at 445, 475, 1147, 1382, 1422, 1494, and 1618 cm^−1^. A LOD of 1 × 10^−12^ M was achieved for the SERS-based detection of MB on the Au-coated ZnO NRs.

In another investigation, a hybrid piezotronic material comprising Au-decorated ZnO NR arrays was developed as a wearable self-powered SERS substrate for the detection of lactate in sweat [6]. Vertically aligned ZnO NRs with an average diameter of ~150 nm and an average length of ~12.5 µm were hydrothermally grown on a flexible Ti substrate, followed by the deposition of Au NPs onto the exposed tips of the NRs via magnetron sputtering. The construct of Au NPs/ZnO NRs on Ti was positioned between two rigid transparent quartz plates, with four pairs of magnets at each corner for applying adjustable pressure forces. As the magnetic flux increased, the attraction forces between the magnets intensified, compressing the composite film. This compression boosted the piezoelectric potential of the ZnO, which enhanced the SERS signal. The hybrid material benefited from the piezoelectric property of ZnO and the LSPR effects of Au, with the asymmetric Au NPs/ZnO NRs configuration further improving the charge separation within the material. For lactate measurements, the piezotronic SERS sensor displayed the characteristic Raman peaks of the analyte at 567, 771, 865, 934, 1051, 1091, 1322, 1368, 1435, and 1453 cm^−1^. The peak at 1091 cm^−1^ was used for quantitative detection, yielding a linear detection range within 3–35 mM and a LOD of 2 mM. In another study, individual ZnO–metal nanohybrid platforms were also demonstrated in a WGM-coupled SERS application [53]. A WGM microcavity fabricated from a ZnO microrod (µR) decorated with Ag NPs was employed for the detection of dopamine. The ZnO-Ag hybrid platform was aimed to produce a synergistic effect between the LSPR from the Ag NPs and the WGM from the ZnO µR to enhance the light–matter interactions in SERS measurements. The SERS signals of dopamine, monitored at various Raman shifts of 675, 780, 1150, 1280, 1350, 1450, 1497, and 1580 cm^−1^, were found to increase with dopamine concentration, yielding a LOD of 1 × 10^−12^ M.

#### 2.7.5. Applications of 2D ZnO Nanomaterials in Surface-Enhanced Raman Sensors

Additionally, ZnO thin films were used in the detection of neopterin and metronidazole (MNZ) [54,55]. It was important to tune the thin film-based SERS substrates for their surface roughness so that the small topographic features on the SERS substrates could function as SERS hotspots. For example, an SERS substrate of Au-coated ZnO thin film was developed for the detection of neopterin [54]. ALD was employed to precisely control the thickness and surface roughness of the ZnO thin film by varying the deposition parameters, such as the temperature and purging time. With 10,000 ALD cycles of 2 s purging at 100 °C, a ZnO film was synthesized to a thickness of 1.4 µm with a high surface roughness of ~400 nm. The ZnO thin film was then sputter-coated with a uniform layer of 80 nm thick Au. The Au-coated ZnO substrate was subsequently employed to detect neopterin, for which its characteristic Raman peaks at 695, 1308, 1578, and 1690 cm^−1^ were monitored. A LOD of 1.4 nM, comparable to 0.8 nM in a commercial ELISA test, was obtained from the SERS-based neopterin detection.

**Figure 12:**
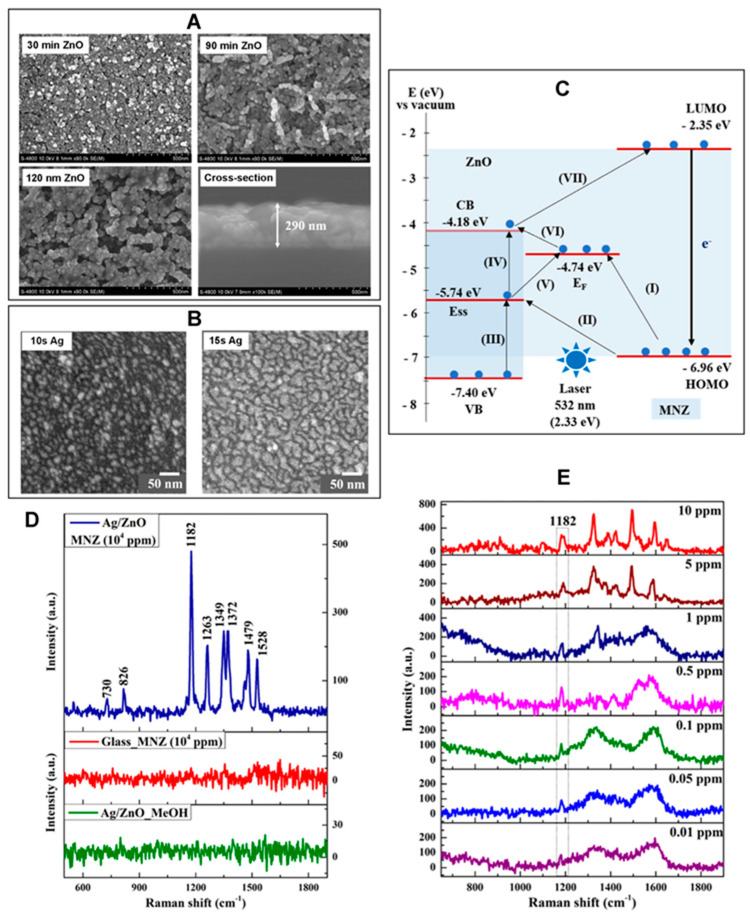
ZnO thin film was used as an SERS biosensor for the detection of MNZ. (**A**) The field emission SEM (FESEM) image corresponds to the 6-layer ZnO thin films prepared by sol-gel dip coating and annealed at 500 °C for 30, 90, and 120 min. The last SEM micrograph is a cross-sectional view of the ZnO thin film annealed for 120 min. (**B**) The FESEM images show the Ag NPs deposited on glass substrates by the DC magnetron sputtering method for 10 and 15 s. (**C**) The schematic diagram depicts the CT processes between the MNZ molecules and the Ag NP-ZnO thin film. When excited by a 532 nm laser, electron movements can occur via multiple pathways between the various energy levels, such as the Fermi energy (E_F_) level of Ag NPs, the VB/CB of ZnO, the surface state energy (Ess) level of ZnO, and the HOMO/LUMO of MNZ. The Ess comes from the surface defects of ZnO. The narrowed optical band gap of the ZnO thin film after annealing can additionally support the CT. The rough and porous thin-film surface also promotes the CT by providing low reflection and high scattering of the incident light. The increased CT processes facilitate SERS through the CE mechanism. (**D**) The plots display the Raman signal of 10^4^ ppm MNZ absorbed on the substrates of (top) the Ag NP-ZnO thin film and (middle) bare glass. The plot in the bottom panel corresponds to the blank solution of methanol (MeOH) deposited on a substrate of Ag NP-ZnO thin film. MeOH was the solvent used to prepare the MNZ solution. The Ag-ZnO substrates were prepared by 10 s sputtering of Ag NPs onto a ZnO thin film annealed for 120 min. (**E**) The plots display the SERS spectra obtained from varying MNZ concentrations absorbed on the Ag NP-ZnO thin film. The Ag/ZnO SERS substrate could detect MNZ at a concentration as low as 0.01 ppm with an SERS EF of ~10^6^. Adapted with permission from Ref. [55], copyright (2023) American Chemical Society.

In another effort, a rough, porous surface of ZnO thin film sputtered with Ag NPs was employed to detect MNZ, as shown in Figure 12A,B [55]. A ZnO thin film was deposited on a glass via six cycles of sol-gel dip coating, followed by thermal annealing at 500 °C for 120 min. Both the dip coating and thermal annealing conditions were optimized to produce a ZnO thin film with a large thickness and a high surface roughness. It was reported that both the carrier concentrations and intrinsic defects in the ZnO substrate increased with the film thickness, which, in turn, provided a higher CT. The annealing time of 120 min, relative to shorter durations, resulted in a high surface area through the formation of cavities and pores in the ZnO film and promoted incident light scattering. A further increase in the annealing time decreased the effective surface area due to the formation of larger grain sizes and reduced porosity. The optimized ZnO thin film was also found to exhibit the smallest band gap. The surface roughness and porosity, as well as the narrow bandgap of the ZnO thin film, were hypothesized to combinedly contribute to a high SERS signal by increasing the scattering of incident light and reducing the reflectance, where both factors promoted CT between the analyte and the substrate. Figure 12C illustrates potential pathways for the CT processes between the different energy levels of Ag NPs, ZnO, and MNZ, which contribute to SERS via the CE mechanism. For EM enhancement, the ZnO thin film was further homogeneously coated with Ag NPs via magnetron sputtering for an optimal condition of 10 s. The resulting Ag NPs on the ZnO thin film exhibited ~20 nm in size, ~5 nm in NP-to-NP distance, and a strong LSPR absorption peak at 433 nm. Both components played an important role in modulating the hotspots for SERS through the EM mechanism. Figure 12D,E present the Raman-scattering data obtained from varying concentrations of MNZ on the SERS substrate of the Ag NP-ZnO thin film, showing a LOD of 0.01 ppm. A characteristic MNZ peak at 1182 cm^−1^ was used to determine the EF enabled on the Ag NP-ZnO thin film, which was found to be 3.30 × 10^6^.

## 3. Conclusions

As detailed in this review, stimulating research advancements have been successfully made in developing various forms of ZnO nanomaterials as novel optical nanobiosensors. ZnO nanomaterial-based biosensors offer a wide range of optical signal transduction modes. Key achievements for the development and application of ZnO nanomaterials are, hence, introduced in this review according to the optical detection modes of absorption, colorimetry, fluorescence, near-band-edge emission, deep-level emission, chemiluminescence, surface evanescence wave, whispering gallery mode, lossy-mode resonance, surface plasmon resonance, and surface-enhanced Raman scattering. This wide variety enables versatility in choosing bioanalyte detection modes tailored to specific bioassay needs, even in complex biomatrices. The majority of cases of ZnO nanomaterials demonstrated so far in optical biosensing rely on the use of ensembles of ZnO nanomaterials and their collective properties. Yet, notable research efforts involving single ZnO NRs and their properties that are distinctively different from those of ensemble behaviors have already begun, as highlighted in this review. This promotes the future prospect of ZnO nanomaterial-based biosensors as highly miniaturized biodetection devices, flexible and wearable biomonitoring gadgets, and non-invasive in vivo probes, as well as nanoscopic subcellular imaging tools. Exploiting the nanoscale dimensions, the use of individual ZnO nanomaterials in such optical biosensing and bioimaging applications can be particularly advantageous to light delivery/collection as well as targeted cargo delivery only to those nanoscopic bioconstituents of interest without affecting other biocomponents.

The main properties of ZnO nanomaterials crucial to the advancements made in each mode of biodetection are explained in the review. Owing to these properties, the presence of ZnO nanomaterials permits significant improvements in important biosensor characteristics. These qualities allow for the better recruitment of bioanalyte molecules, lower LODs, wider linear/dynamic ranges, the elimination of external enzymes or labels, and concentrated optical signals that are highly localized or resonantly coupled. Among the research examples discussed in this review, several cases of bioanalyte detection pertain to the physiological samples of urine, blood, serum, saliva, sweat, and tears. These examples demonstrate that, especially for those widely used detection modes such as fluorescence, the use of ZnO nanomaterials has evolved well beyond the proof-of-concept stage, nearing the goal of their application in disease diagnosis, patient screening, and point-of-care monitoring. As such, the encouraging applications of ZnO nanomaterials in optical biosensing are anticipated to permit ultra-trace-level detection of other bioanalytes and biomarkers in untreated physiological samples, which cannot be readily attained by conventional methods. Commercialization of such ZnO-based sensors can provide new markets for portable, cost-effective diagnostic devices, although challenges such as regulatory approval, scalability, and ensuring stability across various environmental conditions must be addressed to achieve practical implementation. ZnO nanomaterial-based optical biosensors are also expected to significantly advance biomarker discovery, offering deeper insights into disease mechanisms. Moreover, these sensors can facilitate more accurate evaluation of therapeutic interventions, enabling real-time monitoring of disease progression or recurrence. This capability is critical for developing personalized treatment plans and improving patient outcomes. Lastly, it is essential to recognize that these groundbreaking applications are driven by fundamental research, which continues to refine the understanding of nanomaterial properties and their interaction with biological systems, laying the foundation for future innovations in the field of nanobiosensors and nanobiodetection. The future impact of ZnO nanomaterials, hence, can be maximized with unceasing efforts in fundamental research, especially in the areas of the development of novel ZnO nanomaterials, discoveries of unique nanomaterial properties, and hassle-free syntheses of property-tuned nanomaterials.

## Figures and Tables

**Table 1:** The topic of optical biosensors based on ZnO nanomaterials is comprehensively discussed in this Review according to the different optical detection modes, as listed below. The numbers in the brackets correspond to the respective sections covered in the review. For each mode of optical detection, a brief and general overview of the optical mode is followed by the contributions of ZnO nanomaterials specific to the biosensing field. Specific biodetection applications of ZnO nanomaterials are then discussed in detail by grouping the materials by their dimensionality. For simplicity and clarity, all 0D,1D, and 2D forms of nanomaterials will be referred to as NPs, NRs, and thin films, respectively, in this review.

ZnO Nanomaterial Biosensors According to Optical Detection Modes		
**Absorption (Abs)** **and** **Colorimetry (Col)** **[Section 2.1]**	Overview of abs/col in biodetection [Section 2.1.1]	
	Contributions of ZnO nanomaterials in abs-/col-based biodetection [Section 2.1.2]	
	Applications of 0D ZnO NPs in abs/col biosensors [Section 2.1.3]	
	Applications of 1D ZnO NRs in abs/col biosensors [Section 2.1.4]	
**Fluorescence (Fluo)** **[Section 2.2]**	Overview of fluo in biodetection [Section 2.2.1]	
	Contributions of ZnO nanomaterials in fluo-based biodetection [Section 2.2.2]	
	QD fluorescent probes [Section 2.2.2.1]	Fluo signal-enhancing platforms [Section 2.2.2.2]
	Applications of 0D ZnO NPs in fluo biosensors [Section 2.2.3]	
	Applications of 1D ZnO NRs in fluo biosensors [Section 2.2.4]	
	ZnO NRs in fluo-based biodetection [Section 2.2.4.1]	Single ZnO and ZnO-related NRs in fluo-based biodetection [Section 2.2.4.2]
	Applications of 2D ZnO thin films in fluo biosensors [Section 2.2.5]	
**Photoluminescence (PL): Near-band-edge emission (NBE), Deep-level emission (DLE) [Section 2.3]**	Contributions of ZnO nanomaterials in PL-based biodetection [Section 2.3.1]	
	Applications of 0D ZnO NPs in PL biosensors [Section 2.3.2]	
	Applications of 1D ZnO NRs in PL biosensors [Section 2.3.3]	
	Applications of 2D ZnO thin films in PL biosensors [Section 2.3.4]	
**Chemiluminescence (CL)** **[Section 2.4]**	Overview of CL in biodetection [Section 2.4.1]	
	Contributions of ZnO nanomaterials in CL-based biodetection [Section 2.4.2]	
	Applications of 0D/1D ZnO nanomaterials in CL biosensors [Section 2.4.3]	
**Surface evanescent wave (Surface EW), whispering gallery mode (WGM), Lossy-mode resonance (LMR) [Section 2.5]**	Contributions of ZnO nanomaterials in various guided- and lossy-mode biodetection [Section 2.5.1]	
	Applications of 0D/1D ZnO nanomaterials in surface EW biosensors [Section 2.5.2]	
	Applications of 1D ZnO NRs in WGM biosensors [Section 2.5.3]	
	Applications of 2D ZnO thin films in LMR biosensors [Section 2.5.4]	
**Surface plasmon resonance (SPR)** **[Section 2.6]**	Overview of SPR in biodetection [Section 2.6.1]	
	Contributions of ZnO nanomaterials in SPR-based biodetection [Section 2.6.2]	
	Applications of 0D/2D ZnO nanomaterials in SPR biosensors [Section 2.6.3]	
	Applications of 1D ZnO NRs in SPR biosensors [Section 2.6.4]	
**Surface-enhanced Raman scattering (SERS)** **[Section 2.7]**	Overview of SERS in biodetection [Section 2.7.1]	
	Contributions of ZnO nanomaterials in SERS-based biodetection [Section 2.7.2]	
	Applications of 0D ZnO NPs in SERS biosensors [Section 2.7.3]	
	Applications of 1D ZnO NRs in SERS biosensors [Section 2.7.4]	
	Applications of 2D ZnO thin films in SERS biosensors [Section 2.7.5]	

**Table 2:** Optical biodetection studies utilizing ZnO nanomaterials as a key signal transduction component in sensing and quantifying bioanalytes are tabulated. They will be discussed in the specified section under the detection mode. All ZnO applications involve the use of ensembles of ZnO nanomaterials, except for the one marked with (i), which corresponds to the use of individual NRs in biosensing. The detection range is marked either with or without (l) next to the values, indicating the linear and dynamic range of a given biosensor, respectively. Those with N/A reflect that the values were not reported.

Bioanalytes	Nanomaterials	Detection Mode (Section)	Role of ZnO	Detection Range Limit of Detection	Ref.
Lactate	Fe_2_O_3_-ZnO NPs	Abs (Section 2.1.3)	Peroxidase mimic	50–1000 μM (l)	[5]
				9.4 μM	
	AuNPs/ZnO NRs	SERS (Section 2.7.4)	Promoting CE and LSPR excitation, piezotronic enhancement	3–35 mM (l)	[6]
				2 mM	
COVID-19 virus	Chitosan/ZnO NPs/carbon nanotubes (CNTs)	Abs (Section 2.1.3)	Peroxidase mimic	1–500 pg/mL (l)	[7]
				0.05 pg/mL	
		Col (Section 2.1.3)	Peroxidase mimic	50–500 pg/mL (l)	
				8 pg/mL	
Cholesterol	ZnO NPs/CNTs	Col (Section 2.1.3)	Peroxidase mimic	0.5–500 nM (l)	[8]
				0.2 nM	
Glucose	ZnO NPs	PL (Section 2.3.2)	Near-band-edge (NBE) emission source	30–130 mM (l)	[9]
				10 mM	
	ZnO NRs	Col (Section 2.1.4)	Greater enzyme loading	0.01–10 mM (l)	[10]
				3 µM	
		Fluo (Section 2.2.4.1)	Greater CdSe/ZnS QD loading	0.03–3 mM (l)	[11]
				N/A	
	ZnO NRs/ Au NPs	PL (Section 2.3.3)	NBE emission source	0.01–2 mM (l)	[12]
				0.01 mM	
	ZnO/ZnS NRs	PL (Section 2.3.3)	NBE emission source	3.5–24 mM (l)	[13]
				0.14 mM	
	ZnO NRs/ Ag film	SPR (Section 2.6.4)	Greater enzyme binding, high index material	0–10 mM	[14]
				0.012 mM	
Uric Acid	ZnO NRs	Col (Section 2.1.4)	Greater enzyme loading	0.01–5 mM (l)	[10]
				4 µM	
		Surface EW (Section 2.5.2)	Surface evanescence generation	0–500 ppm (l)	[15]
				5.6 ppm	
Cysteine	Melamine/ ZnO QDs	Fluo (Section 2.2.3)	QD fluorophore	0.1–600 µM (l)	[16]
				0.642 µM	
Calcium dipicolinate	Eu/ZnO QDs	Fluo (Section 2.2.3)	QD fluorophore	0–4 µM (l)	[17]
				3 nM	
Tetracycline	Eu/ZnO QDs	Fluo (Section 2.2.3)	QD fluorophore	5 nM–3 µM (l)	[18]
				4 nM	
40 base-pair-long DNA	ZnO/CdS QDs	Fluo (Section 2.2.3)	FRET donor	75.82 pM–15.28 nM (l)	[19]
				8.289 pM	
Avidin	Mn-doped ZnS/ZnO QDs	Fluo (Section 2.2.3)	Higher quantum yield	10–100 nM (l)	[20]
				3 nM	
Immunoglobulin G antibodies	ZnO NRs	Fluo (Section 2.2.4.1)	Signal-enhancing platform	N/A	[21]
		Fluo (Section 2.2.4.2)	Signal-enhancing platform	N/A	[22,23]
*Bacillus anthracis* DNA	ZnO NRs	Fluo (Section 2.2.4.1)	Signal-enhancing platform	N/A	[24]
Immunoglobulin G	ZnO NPs coated with Au	SPR (Section 2.6.3)	Greater biomolecule loading	0.0375–40 µg/mL	[25]
				0.0375 µg/mL	
	ZnO NRs	Fluo (Section 2.2.4.1)	Signal-enhancing platform	N/A	[26]
Bovine serum albumin	CdSe/ZnO QDs	SERS (Section 2.7.3)	Promoting chemical enhancement (CE)	N/A	[27]
				2.5 × 10^−6^ M	
	ZnO NRs	Fluo (Section 2.2.4.1)	Signal-enhancing platform	N/A	[26]
Telomerase	ZnO NRs	Fluo (Section 2.2.4.1)	Signal-enhancing platform	N/A	[28]
Interleukin-8	ZnO NRs	Fluo (Section 2.2.4.1)	Signal-enhancing platform	10 fg/mL–10 ng/mL (l)	[29]
				5.5 fg/mL	
Tumor necrosis factor-α	ZnO NRs	Fluo (Section 2.2.4.1)	Signal-enhancing platform	10 fg/mL–1 ng/mL (l)	[29]
				4.2 fg/mL	
	ZnO NR (i)	Fluo (Section 2.2.4.2)	Subwavelength waveguide	N/A	[30]
Rheumatoid arthritis autoantibodies	ZnO NRs	Fluo (Section 2.2.4.1)	Signal-enhancing platform	N/A	[31]
Adenosine triphosphate	ZnO NRs	Fluo (Section 2.2.4.1)	Signal-enhancing platform	1 pM–100 µM (l)	[32]
				1 pM	
Cardiac troponin I	Magnetic beads-ZnO NRs	Fluo (Section 2.2.4.1)	Signal-enhancing platform	N/A	[33]
				252.4 pg/mL	
Carcinoembryonic antigen	ZnO NPs	CL (Section 2.4.3)	Catalyzing radical and electron processes in CL	0.001–20 ng/mL (l)	[34]
				0.001 ng/mL	
	ZnO NRs	Fluo (Section 2.2.4.1)	Signal-enhancing platform	N/A	[35]
				10 fg/mL	
		Fluo (Section 2.2.4.1)	Greater biomolecule loading, FRET acceptor	0.001–100 ng/mL	[36]
				0.001 ng/mL	
Green fluorescent protein	ZnO/Ag/PMMA thin film	Fluo (Section 2.2.5)	Greater antibody loading	10 pM–100 nM	[37]
				7 pM	
Soluble epidermal growth factor receptor	ZnO/Ag/PMMA thin film	Fluo (Section 2.2.5)	Greater antibody loading	700 fM–10 nM	[38]
				700 fM	
*N*-acyl homoserine lactone	Cysteamine/ ZnO NPs	PL (Section 2.3.2)	Deep-level emission (DLE) source	10–120 nM (l)	[39]
				N/A	
Trypsin	Diphenylalanine/ZnO NPs	PL (Section 2.3.2)	DLE source	0–160 ng/mL (l)	[40]
				0.1 ng/mL	
Calf thymus DNA	ZnO/Au NPs	PL (Section 2.3.2)	DLE source	0.1–0.7 µM (l)	[41]
				36 nM	
*Escherichia coli* DNA	ZnO NRs	PL (Section 2.3.3)	DLE source	0.102–0.894 µM	[42]
				28.4 nM	
Ochratoxin A	ZnO NRs	PL (Section 2.3.3)	NBE emission source	0.1–1 ng/mL	[43]
				0.1 ng/mL	
Grapevine virus A-type proteins	Mn-doped ZnO NRs	WGM (Section 2.5.3)	WGM resonator	1–200 ng/mL	[44]
				N/A	
	ZnO thin film	PL (Section 2.3.4)	DLE source	0.001–10 ng/mL	[45]
				N/A	
Choline	ZnO NRs	CL (Section 2.4.3)	Greater enzyme loading	0.006–2 mM (l)	[46]
				0.0005 mM	
Urea	Polyaniline/ ZnO NPs	Surface EW (Section 2.5.2)	Surface evanescence generation	10 nM–1 M (l)	[47]
				10 nM	
Cortisol	Polypyrrole/ ZnO thin film	LMR (Section 2.5.4)	Lossy-mode resonator	10–10^5^ pg/mL (l)	[48]
				25.9 fg/mL	
p-Cresol	ZnO/MoS_2_ thin film	LMR (Section 2.5.4)	Lossy-mode resonator	0.028–1000 µM	[49]
				28 nM	
*Neisseria meningitidis* DNA	ZnO thin film/Au	SPR (Section 2.6.3)	Greater DNA loading, high-index material	10–180 ng/µL (l)	[50]
				5 ng/µL	
Cellular DNA, lipids, proteins	ZnO NPs	SERS (Section 2.7.3)	CE, greater analyte binding	N/A	[51]
SARS-CoV-2 spike protein	AuNPs/ZnO NRs/Ag film	SERS (Section 2.7.4)	Promoting LSPR excitation	N/A	[52]
				0.36 (1.6) × 10^−16^ M in PBS (saliva)	
Dopamine	AgNPs/ ZnO microrods (i)	SERS (Section 2.7.4)	WGM resonator	N/A	[53]
				1 × 10^−12^ M	
Neopterin	Au-coated ZnO thin film	SERS (Section 2.7.5)	Promoting CE and LSPR excitation	N/A	[54]
				1.4 nM	
Metronidazole	Ag-coated ZnO thin film	SERS (Section 2.7.5)	Promoting CE and LSPR excitation	N/A	[55]
				0.01 ppm	
